# Resolving the source of branch length variation in the Y chromosome phylogeny

**DOI:** 10.1186/s13059-024-03468-4

**Published:** 2025-01-06

**Authors:** Yaniv Swiel, Janet Kelso, Stéphane Peyrégne

**Affiliations:** Department of Evolutionary Genetics, Max Planck Institute for Evolutionary Anthropology, Leipzig, Germany

**Keywords:** Reference bias, Ancient DNA, Y chromosome, Sequence alignment, Molecular dating, Mutation rate, Generation time

## Abstract

**Background:**

Genetic variation in the non-recombining part of the human Y chromosome has provided important insight into the paternal history of human populations. However, a significant and yet unexplained branch length variation of Y chromosome lineages has been observed, notably amongst those that are highly diverged from the human reference Y chromosome. Understanding the origin of this variation, which has previously been attributed to changes in generation time, mutation rate, or efficacy of selection, is important for accurately reconstructing human evolutionary and demographic history.

**Results:**

Here, we analyze Y chromosomes from present-day and ancient modern humans, as well as Neandertals, and show that branch length variation amongst human Y chromosomes cannot solely be explained by differences in demographic or biological processes. Instead, reference bias results in mutations being missed on Y chromosomes that are highly diverged from the reference used for alignment. We show that masking fast-evolving, highly divergent regions of the human Y chromosome mitigates the effect of this bias and enables more accurate determination of branch lengths in the Y chromosome phylogeny.

**Conclusion:**

We show that our approach allows us to estimate the age of ancient samples from Y chromosome sequence data and provide updated estimates for the time to the most recent common ancestor using the portion of the Y chromosome where the effect of reference bias is minimized.

**Supplementary Information:**

The online version contains supplementary material available at 10.1186/s13059-024-03468-4.

## Introduction

Human Y chromosome lineages that are highly diverged from the reference Y chromosome appear to have accumulated fewer mutations than lineages that are more closely related to the reference, a non-African lineage of the R1b haplogroup. In particular, some human Y lineages in Africa, such as A00 [1], appear to have accumulated fewer mutations than other Y lineages since they last shared a common ancestor.

In their phylogenetic analysis of a diverse selection of human Y chromosomes for worldwide populations, Wei et al. [2] observed a shortened branch for the A3 haplogroup represented by a single individual in their study. They attributed this shortened branch to an under-calling of variants due to the low-coverage sequencing (5×) of the A3 individual. Scozzari et al. [3] analyzed Y chromosomes representing major haplogroups and noted shortened branches for the A haplogroups relative to the rest of the tree. They were not able to determine the cause of the shortened branches, but suggested that a reduction in the male effective population size coinciding with the out-of-Africa bottleneck allowed mildly deleterious mutations to accumulate on non-African Y chromosomes. Hallast et al. [4] tested whether the tissue from which the DNA is extracted has an effect on the branch length heterogeneity they observed but could not detect any statistically significant differences between tissues. They therefore proposed that a change in the mean paternal age (resulting in the increase or decrease of the effective mutation rate) in a particular geographical region could result in associated haplogroups displaying similarly shortened or lengthened branches. Barbieri et al. [5] also hypothesized that population differences in average paternal age could contribute to the branch length variation they observed across the Y chromosome phylogeny.

Barbieri et al. [5] additionally suggested a number of technical biases that could result in the undercalling of variants in A and B Y chromosome lineages. They proposed that capture bias could be introduced when using a Y chromosome capture array based on the reference Y chromosome and that the underrepresentation of A and B haplogroups in reference datasets could affect genotype calling and/or imputation. Although these studies relied on capture sequencing data (and low-coverage shotgun sequencing data in the case of Wei et al. [2]), Naidoo et al. [6] also noticed shortened branches among A haplogroups using high-coverage shotgun sequencing data, suggesting that these differences do not originate from capture bias.

Understanding the cause of branch length variation in the Y chromosome phylogeny is important to accurately reconstruct human evolutionary history. The A00 Y chromosome lineage has been used to estimate the time to the most recent common ancestor (TMRCA) of the Y chromosomes of all living humans [7] and studies of archaic human Y chromosomes [1, 8] have used this TMRCA to estimate the TMRCA between modern and archaic human Y chromosomes. The branch length variation observed suggests that there may be differences in mutation rate among Y chromosome lineages. Therefore, incorrectly assuming a constant mutation rate over the Y chromosome phylogeny could result in biased TMRCA estimates that do not accurately reflect population split times. Moreover, variation in the Y chromosome mutation rate could bias methods that estimate the age of ancient and archaic humans by comparing the number of mutations accumulated on their Y chromosomes to those of present-day human Y chromosomes.

Newly sequenced, high-quality Y chromosomes from present-day [7, 9, 10] and ancient modern humans [11–14], as well as from Neandertals [1, 15], provide an opportunity to re-evaluate the hypotheses that changes in mutation rate or generation time are the cause of the branch length variation in the Y chromosome phylogeny. These Y chromosomes also allow us to test the effect of reference bias on the mapping of short reads. Reference bias refers to the observation that sequencing reads carrying non-reference alleles are less likely to align successfully to the reference genome than those carrying reference alleles. The effect of reference bias is exacerbated for shorter read lengths and for reads carrying ancient DNA damage, which introduces more mismatches to the reference genome [16, 17]. Y chromosomes that are divergent from the reference Y chromosome may be particularly affected by reference bias, making it a possible explanation for the branch shortening reported for some African Y chromosome lineages. The recent availability of high-quality Y chromosome assemblies—including a telomere-to-telomere Y chromosome assembly [18] and an almost fully contiguous assembly of a Y chromosome representing the basal A0b haplogroup [19]—allow us to test whether the reference used for alignment has any effect on branch length.

## Results

### Branch length variation among a diverse set of Y chromosomes

**Fig. 1:**
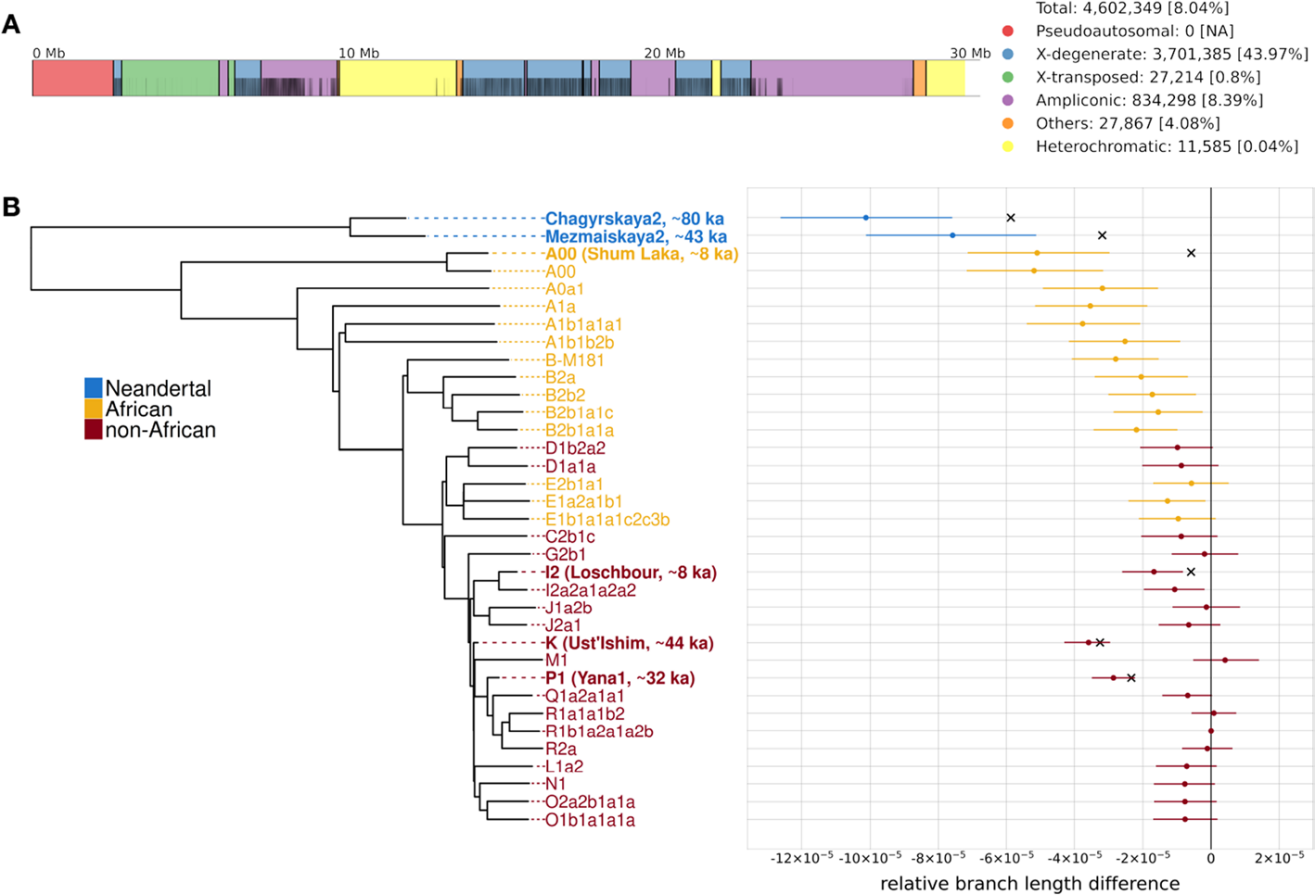
**a** Structure of the hg19 Y chromosome showing the different sequence classes using coordinates from [20]. The black vertical lines represent the uniquely mappable positions used for the analysis (see the “Materials and methods” section). The legend indicates the number of uniquely mappable positions and the proportion of each sequence class included in the analysis. **b** Neighbor-joining tree of a Y chromosome phylogeny comprising two Neandertals [1, 15], four ancient humans [11–14] and twenty nine present-day humans [7, 9, 10] with associated relative branch length differences (i.e., the difference in branch length normalized by the total number of sites used for the comparison) compared to a present-day non-African Y chromosome (R1b1a2a1a2b). The haplogroup names are taken from the respective publications. The colors indicate the population of origin. The ancient individuals and their estimated ages are marked in bold. The crosses denote the expected relative branch length differences for the ancient individuals based on their estimated ages and assuming a constant mutation rate of $7.34 \times 10^{-10}$ mutations/bp/year [1]. The error bars represent 95% confidence intervals (CIs) computed by resampling branch lengths from a Poisson distribution as described in Petr et al. [1]

To understand why there appear to be fewer mutations in Y chromosomes that are diverged from the reference used for alignment, we compiled a dataset comprising 35 high-quality Y chromosomes from present-day and ancient modern humans, including those from two Neandertals (Additional file 1: Table S1). We based our analyses on the $\sim$4.6 Mb of the non-recombining portion of the Y chromosome that is uniquely mappable (Fig. 1a, see the “Materials and methods” section). While the dataset contains Y chromosomes representing all of the major haplogroups, our focus was on incorporating Y chromosomes that are significantly diverged from the human reference (hg19, haplogroup R1b) Y chromosome. To determine whether there is branch length variation in our dataset, we compared the number of apparent mutations on each Y chromosome to the number in a present-day non-African Y chromosome (haplogroup R1b1a2a1a2b) since their common ancestor, thereafter referred to as relative branch length difference. If the mutation rate was constant across the Y chromosome phylogeny, we would expect no significant differences between the number of mutations that we detect on the Y chromosomes of present-day individuals. However, significantly fewer mutations are identified for those African Y chromosomes that are the most diverged from the reference, and this difference increases linearly with increasing divergence from the reference (Fig. 1b). We find that $\sim$52 mutations are missed with each additional pairwise difference from the reference per 10,000 basepairs (bp) (Additional file 1: Fig. S1).

We expect to observe shorter branches for ancient individuals than for modern individuals because they have less time to accumulate mutations. This explains the shorter branches for *Ust’Ishim* and *Yana1* (Fig. 1b). However, we would expect that ancient individuals with similar radiocarbon dates should have branch lengths similar to one another. Instead, when we compare the Y chromosomes of ancient individuals with similar radiocarbon dates but with differing levels of divergence from the reference (i.e., *Mezmaiskaya2* vs *Ust’Ishim*/*Shum Laka* vs *Loschbour*), we find that the divergent branches are significantly shorter than the non-divergent branches. As variation in branch lengths among African Y chromosomes correlates with divergence from the reference ($r = -0.916$ , $P = 1.072 \times 10^{-5}$, Fig. 1b, Additional file 1: Fig. S1), this is unlikely to be the result of an accumulation of mutations on non-African Y chromosomes following the out-of-Africa bottleneck as proposed by Scozzari et al. [3].

### Testing the hypothesis of mutation rate and generation time variation

The significant branch length variation observed over the Y chromosome phylogeny suggests that the assumption of a constant Y chromosome mutation rate and constant generation time may be violated. Therefore, we next estimated (1) the change in mutation rate (assuming a constant generation time) and (2) the change in generation time (assuming a constant mutation rate) required to explain the observed differences in branch lengths.

**Fig. 2:**
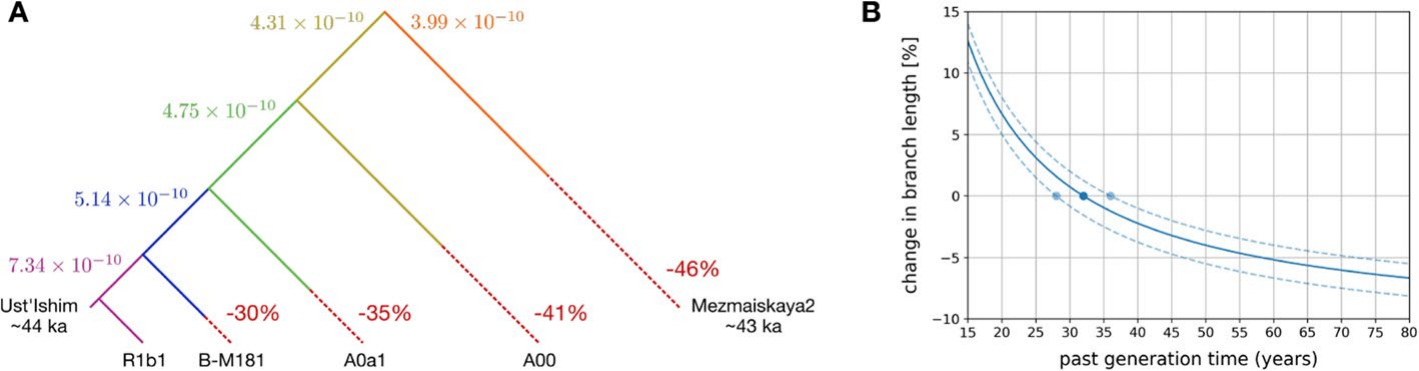
**a** Mutation rates (in units of mutations/bp/year) required to explain the branch lengths of the Y chromosomes that are highly diverged from the human reference. The dashed lines and percentages represent the branch shortening relative to the R1b1a2a1a2b branch (or the expected length given the age for *Mezmaiskaya 2*). The colors indicate the branches of the tree and their corresponding mutation rate. **b** Percentage change in branch length as a function of the past male generation time (*x*-axis) compared to the branch length assuming a male generation time of 32 years, a large value within the range of the estimated male generation time in modern populations [22]. The dashed lines correspond to the change in branch length assuming a generation time of 28 or 36 years

To estimate the change in mutation rate that would result in the observed branch length differences, we compared the branch lengths of the Y chromosomes that are the most diverged from the reference to that of an R1b1 Y chromosome, which is closely related to the hg19 reference (R1b). For the Neandertal Y chromosome (*Mezmaiskaya 2*), we compared the branch length to that expected based on the age of this individual and assuming a Y chromosome mutation rate of $7.34 \times 10 ^{-10}$ mutations/bp/year [1], which was computed by comparing the difference in the number of mutations between an ancient human Y chromosome (that of a 44.5 thousand year old Eurasian hunter-gatherer, *Ust’Ishim* [11]) and those of present-day non-Africans. We find that the branches of the divergent Y chromosome lineages are between 30% and 46% shorter than expected for the least and most divergent lineages, respectively (Fig. 2a and Additional file 1: Table S2). This suggests that multiple changes in mutation rate are needed to explain the observed branch shortening, and that the mutation rate would need to have been up to 46% slower than that estimated in non-Africans. This contrasts with the finding, based on a comparison between the complete assemblies of human and chimpanzee Y chromosomes, that the chimpanzee Y chromosome mutation rate is 11% faster than that of humans [21]. Mutation rate differences alone are, therefore, unlikely to explain the observed differences in branch lengths.

We then tested whether changes in male generation time (i.e., in the average paternal age) could contribute to the observed branch length variation using the relationship between paternal age and the number of autosomal *de novo* mutations as a proxy for *de novo* mutations on the Y chromosome (see the “Materials and methods” section). While the number of *de novo* mutations increases linearly with increasing paternal age [23] (Additional file 1: Fig. S2a), a shorter generation time can result in more mutations because more generations occur over time (Additional file 1: Fig. S2b). We find that no reasonable change in male generation time could account for the apparent 46% shorter branch for the Neandertal lineage, as a change in male generation time could result in, at most, a 10% branch shortening (Fig. 2b).

### Exploring alternative hypotheses for the origin of branch length variation

Since neither biological nor demographic factors fully explain the branch length variation in the human Y chromosome phylogeny, we attempted to determine the extent to which reference bias could cause us to undercount mutations in Y chromosomes that are diverged from the reference used for alignment.

#### Mapping to the chimpanzee reference Y chromosome

We first tested whether the use of an outgroup Y chromosome (that of the chimpanzee) as a reference can correct for the branch length variation in the human Y chromosome phylogeny. Although this strategy should result in a significant level of reference bias for all human Y chromosomes, we expect this bias to be the same across all lineages, as all are equally diverged from the chimpanzee Y chromosome reference. As we obtained many heterozygous genotype calls with the alignments to the chimpanzee reference (suggesting a high frequency of mapping errors) we concluded that human Y chromosomes are too diverged from the chimpanzee Y chromosome to allow us to accurately call genotypes (Additional file 1: Table S3).

#### Mapping to different human Y chromosome assemblies

Due to the highly repetitive nature of the human Y chromosome, more than half of the hg19 reference Y chromosome could not be assembled using short read sequencing methods [18]. A fully contiguous telomere-to-telomere (T2T) Y chromosome assembly [18] (haplogroup J1a2b) allows us to test whether the use of a higher-quality reference influences the number of mutations we are able to detect by facilitating the alignment of more sequences in complex regions. To test whether we can recover more mutations from the more basal lineages in the phylogeny, we also used a Y chromosome reference that is outside of the variation of most human haplogroups (haplogroup A0b [19]).

To study the effect of the reference used for alignment and determine whether reference bias affects mapping to the Y chromosome, we aligned whole-genome sequence data from the individuals in our dataset for whom we had unmapped reads to three different reference genomes:

- The hg19 reference with the original hg19 Y chromosome, which is almost entirely derived from a lineage that represents the R1b haplogroup [24, 25] (one that is within the variation of present-day non-African Y chromosomes)
- The hg19 reference with a long-read assembled Y chromosome representing the A0b lineage [19] (one that is outside of the variation of most modern human Y chromosome lineages, with only the A00 lineage as a more basal outgroup)
- The hg19 reference with the long-read assembled T2T Y chromosome representing the J1a haplogroup [18] (which, like the hg19 Y chromosome, is within the variation of present-day non-African Y chromosomes)

**Fig. 3:**
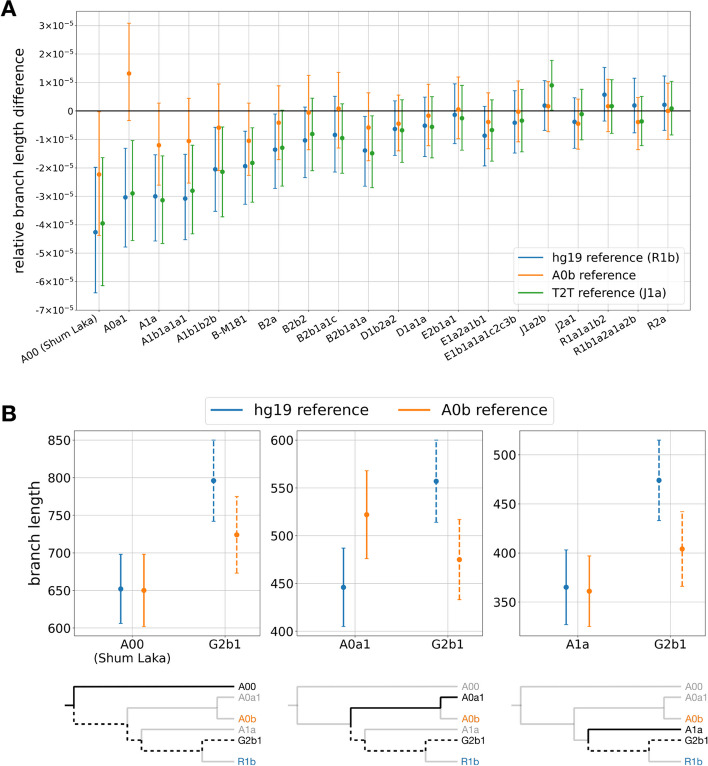
**a** Relative branch length differences compared to a present-day non-African lineage (G2b1) using variants called from alignments to three different reference genomes (shown with different colors). The error bars correspond to 95% CIs computed by resampling branch lengths from a Poisson distribution. **b** Branch lengths for three present-day A lineages (A00, A0a1, and A1a, as indicated in each panel) since their last common ancestor with a present-day non-African lineage (G2b1) using variants called from alignments to two different reference genomes (shown with different colors). The solid lines represent the branch length of the respective A lineage and the dashed lines represent the branch length of the G2b1 lineage. The results with the alignments to the T2T reference genome are similar to those obtained with the hg19 reference, and are not shown for simplicity

For each of the three reference genomes, we processed our dataset using the same pipeline, called genotypes, and compared the relative branch length differences with respect to a present-day non-African Y chromosome (Fig. 3a). In order to compare the effect of the T2T reference against the hg19 reference, we used the G2b1 haplogroup as it is an outgroup to both the J (T2T) and R (hg19) branches of the phylogeny. We find that the T2T reference has little effect on the branch length variation and we observe similar branch length differences for the two references that are within the variation of non-African Y chromosomes. This shows that mutations are not missed due to the quality of the reference assembly. By contrast, the A0b reference enables the detection of more mutations on the A0a1 lineage, but does not resolve the issue for the other A haplogroups that do not fall on the A0 lineage. We also miss mutations on the non-African lineages that are now highly diverged from the A0b reference (Fig. 3b). Taken together, these results show that reference bias does affect the alignment of sequences from human Y chromosomes and that this bias will always be present for some lineages in the phylogeny depending on the reference used for alignment.

### An approach to limit the effect of reference bias

**Fig. 4:**
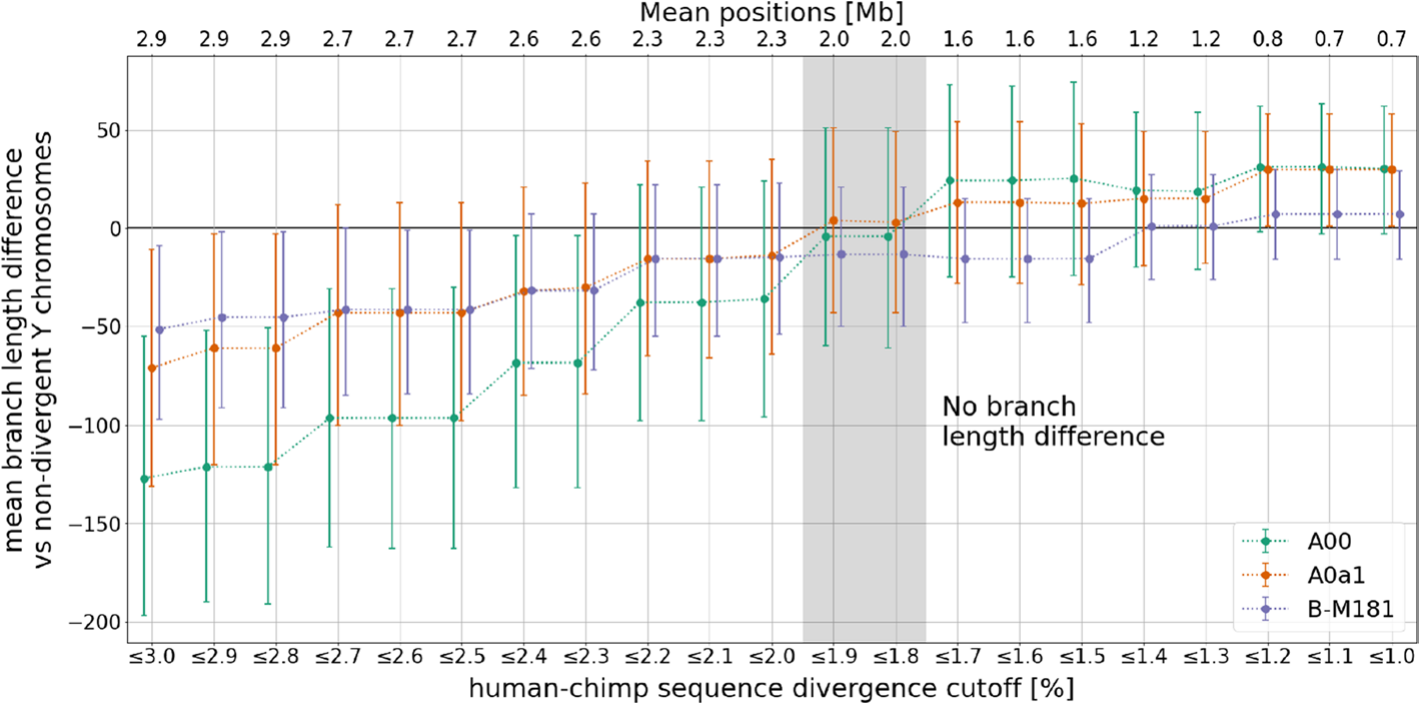
Comparing the number of mutations on divergent Y chromosomes to non-divergent Y chromosomes for different maximum human-chimp sequence divergence filters (lower *x*-axis). The upper *x*-axis represents the average number of positions (in Mb) used for each comparison. The colors correspond to the three divergent Y chromosomes (A00, A0a1, and B-M181). The shaded area indicates the divergence filters that minimize the branch length variation while maximizing the proportion of the Y chromosome available for further analysis. The error bars denote 95% CIs computed by resampling branch lengths from a Poisson distribution

As the reference used for alignment cannot resolve the effect of reference bias for all Y chromosome haplogroups, we then investigated whether specific regions of the hg19 Y chromosome are correlated with a reduction in the ability to call private mutations in divergent Y chromosomes. We used sequence divergence between the hg19 Y chromosome assembly and the chimpanzee reference (panTro6) Y chromosome assembly to identify the faster-evolving regions of the human Y chromosome where the alignment of short read data may be most affected by reference bias. We used a sliding window approach to compute the sequence divergence for each uniquely mappable base of the hg19 Y chromosome based on the number of mismatches compared to the panTro6 Y chromosome. For three present-day haplogroups that are affected by reference bias (A00, A0a1, and B-M181), we computed the mean mutation count difference with respect to all of the haplogroups that do not show significant branch length differences (i.e., all non-African haplogroups along with the E haplogroups) for progressively increasing sequence divergence filters (Fig. 4). This allowed us to identify the sequence divergence cutoff that minimizes the branch length difference between the lineages that are most diverged from the reference and those that are closer to the reference. As the non-recombining part of the Y chromosome is a single locus, only one phylogeny exists for the human Y chromosome. For this reason, removing regions of the Y chromosome does not introduce biases if we use the appropriate mutation rate in subsequent analyses.

Yet, there is a tradeoff between filtering the regions of the Y chromosome where mapping is more difficult while simultaneously retaining a sufficient number of positions for further phylogenetic analysis, and we aimed to retain as many of the uniquely mappable positions of the human Y chromosome as possible. The branch length variation is minimized, and the number of positions maximized, using a human-chimp sequence divergence cutoff of 1.9% which retains around 2 Mb of the uniquely mappable $\sim$4.6 Mb of the hg19 Y chromosome.

We also looked at human-chimp sequence divergence for the complete nuclear genome to determine whether reference bias could have a similar impact on analyses of the autosomes or the X chromosome (Additional file 1: Table S4). The Y chromosome stands out for both the proportion of the chromosome that can be aligned and the divergence from the chimpanzee reference, suggesting that reference bias is largest on the Y chromosome. This probably results from the faster evolving nature of the Y chromosome compared to the autosomes [11].

We then analyzed the regions of the Y chromosome that are removed by the human-chimp divergence filter. About half of the non-recombining part of the human Y chromosome consists of highly repetitive heterochromatin and the remaining euchromatin comprises three sequence classes: the X-degenerate regions, the X-transposed regions, and the ampliconic regions, which primarily consist of palindromic repeats. We find that the majority of the uniquely mappable positions in the ampliconic and X-transposed regions are removed by the divergence filter, whereas more than half of the X-degenerate positions are retained (Table 1). We investigated whether the divergence filter is required in the X-degenerate regions of the Y chromosome by using only these regions when testing for the presence of branch length variation. We still observe shortened branches for the Y chromosome lineages that are most diverged from the reference (Additional file 1: Fig. S3) showing that reference bias also affects the X-degenerate regions and that the divergence filter is necessary for unbiased analyses of the human Y chromosome phylogeny.

**Table 1:** Uniquely mappable positions that pass the human-chimp divergence filter for each euchromatic sequence class of the non-recombining portion of the Y chromosome

Sequence class	Uniquely mappable	Human-chimp aligned	Retained after divergence filtering
X-degenerate	3,701,385	3,476,366 [94%]	2,151,164 [58%]
X-transposed	27,214	0	NA
Ampliconic	834,298	336,588 [40%]	38,196 [5%]
Others	39,452	37,152 [94%]	11,458 [29%]
**Total**	**4,602,349**	**3,850,106 [84%]**	**2,200,818 [48%]**

The percentages represent the proportion of uniquely mappable positions retained

We hypothesized that regions masked by the divergence filter would show evidence for an increased rate of mapping errors. To assess this possibility we identified heterozygous sites called by snpAD—a diploid genotype caller—on the haploid Y chromosome, and calculated the percentage change in the number of heterozygous calls for each individual after applying the human-chimp sequence divergence filter. We found that a large proportion (between 7 and 92%) of mapping errors occur in the divergent regions of the Y chromosome (Additional file 1: Table S5), indicating the increased difficulty of correctly aligning reads in quickly-evolving regions.

### Application to estimates of the modern human and the modern human-Neandertal Y chromosome TMRCAs

**Fig. 5:**
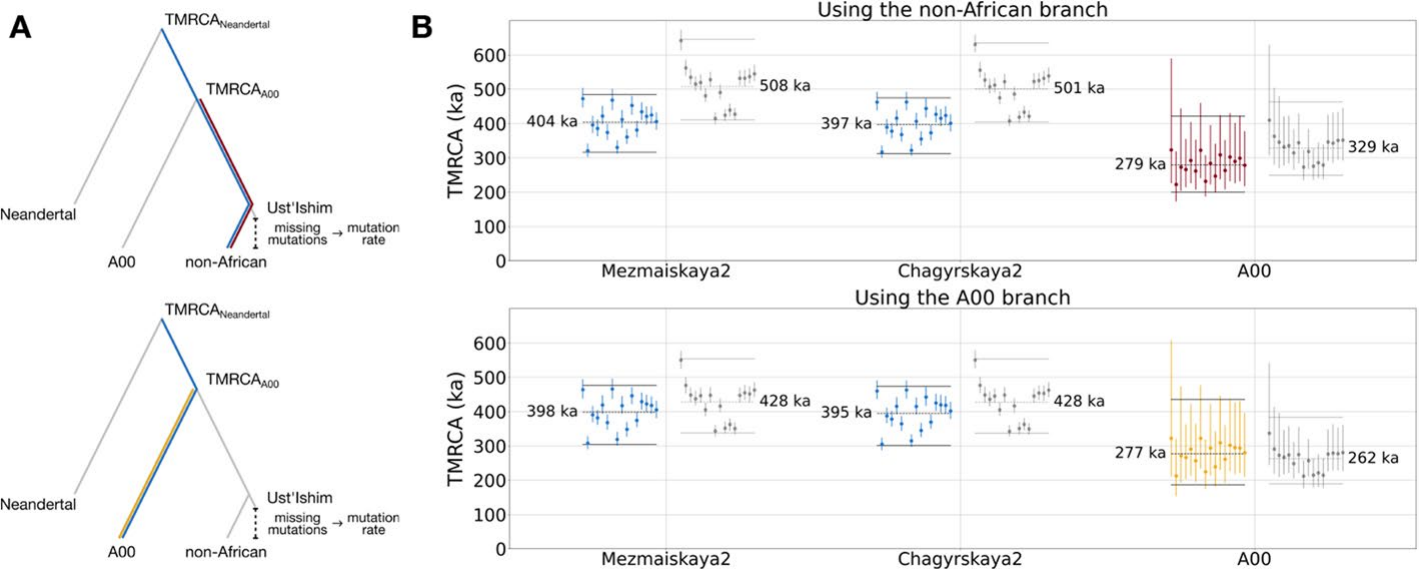
**a** Trees depicting the lineages used to estimate the TMRCA of all modern humans and the TMRCA of modern humans and Neandertals. The lineage of the radiocarbon dated ancient modern human, *Ust’Ishim*, used to calculate the mutation rate, is also shown. **b** TMRCA estimates between the Y chromosomes on the *x*-axis and 16 non-African Y chromosomes. Each dot represents the TMRCA with one non-African Y chromosome. The top plot shows the TMRCAs estimated by measuring the non-African branch, while the bottom plot shows the TMRCAs estimated by measuring the A00 branch. The dots in color represent the TMRCA estimates based on the the filtered Y, while the dots in gray represent the TMRCAs estimated using the unfiltered Y chromosome. The vertical lines denote 95% CIs computed by resampling branch lengths from a Poisson distribution. The dashed horizontal lines represent the mean TMRCAs computed over all non-African Y chromosomes and the solid horizontal lines show overall 95% CIs

After minimizing the effect of reference bias in the Y chromosome phylogeny, we sought to recalibrate both the modern human Y chromosome TMRCA (using the A00 Y chromosome to represent the most basal modern human lineage) and the modern human-Neandertal Y chromosome TMRCA using the portion of the human Y chromosome with minimal reference bias. We used two different methods to determine the Y chromosome TMRCAs: (1) the method defined in [1] (with the lineages used shown in Fig. 5a) which computes the modern human-Neandertal TMRCA by scaling the modern human TMRCA based on modern human-Neandertal divergence and (2) Bayesian phylogenetic reconstruction as implemented in the software package BEAST2 (v2.6.7) [26].

We first calculated the Y chromosome mutation rate for those regions of the Y chromosome that pass the divergence filter (Table 2). The mutation rate in these regions is slightly lower than the mutation rate computed over the entire uniquely mappable Y chromosome (Table 2) and the rates reported elsewhere (Additional file 1: Table S6). This is expected as more divergent, faster-evolving regions of the Y chromosome are removed by the divergence filter. In all subsequent analyses, we used this recalibrated mutation rate to account for the effect of the divergence filter.

**Table 2:** Comparing the mutation rate estimates (in units of mutations/bp/year) for the uniquely mappable ($\sim$4.6 Mb) part of the Y chromosome and for the uniquely mappable, divergence-filtered Y chromosome (maximum 1.9% human-chimp sequence divergence, $\sim$2 Mb)

Method	Unfiltered Y chromosome	Divergence-filtered Y chromosome
Branch shortening	$7.24 \times 10 ^ {-10}$	$6.61 \times 10 ^ {-10}$
	95% CI $4.90 \times 10 ^ {-10}$ to $9.77 \times 10 ^ {-10}$	95% CI $4.12 \times 10 ^ {-10}$ to $9.55 \times 10 ^ {-10}$
BEAST	$7.45 \times 10 ^{-10}$	$6.79 \times 10 ^{-10}$
	95% HPD $6.85 \times 10 ^ {-10}$ to $8.00 \times 10 ^ {-10}$	95% HPD $6.07 \times 10 ^ {-10}$ to $7.54 \times 10 ^ {-10}$

The method used to estimate the mutation rate (as described in the main text) is indicated. HPD - highest posterior density

We then computed the modern human and the modern human-Neandertal TMRCAs, and compared the TMRCAs estimated with all of the uniquely mappable sites to those estimated with only sites that pass the divergence filter (Fig. 5). When the unfiltered Y chromosome is used, the choice of the modern human branch used for comparison has an effect on the TMRCA calculation with the difference in length between the A00 branch and the non-African branches resulting in inconsistent estimates. By applying the divergence filter, the effect of reference bias is minimized and the choice of modern human branch then has no effect on the TMRCA estimates.

**Fig. 6:**
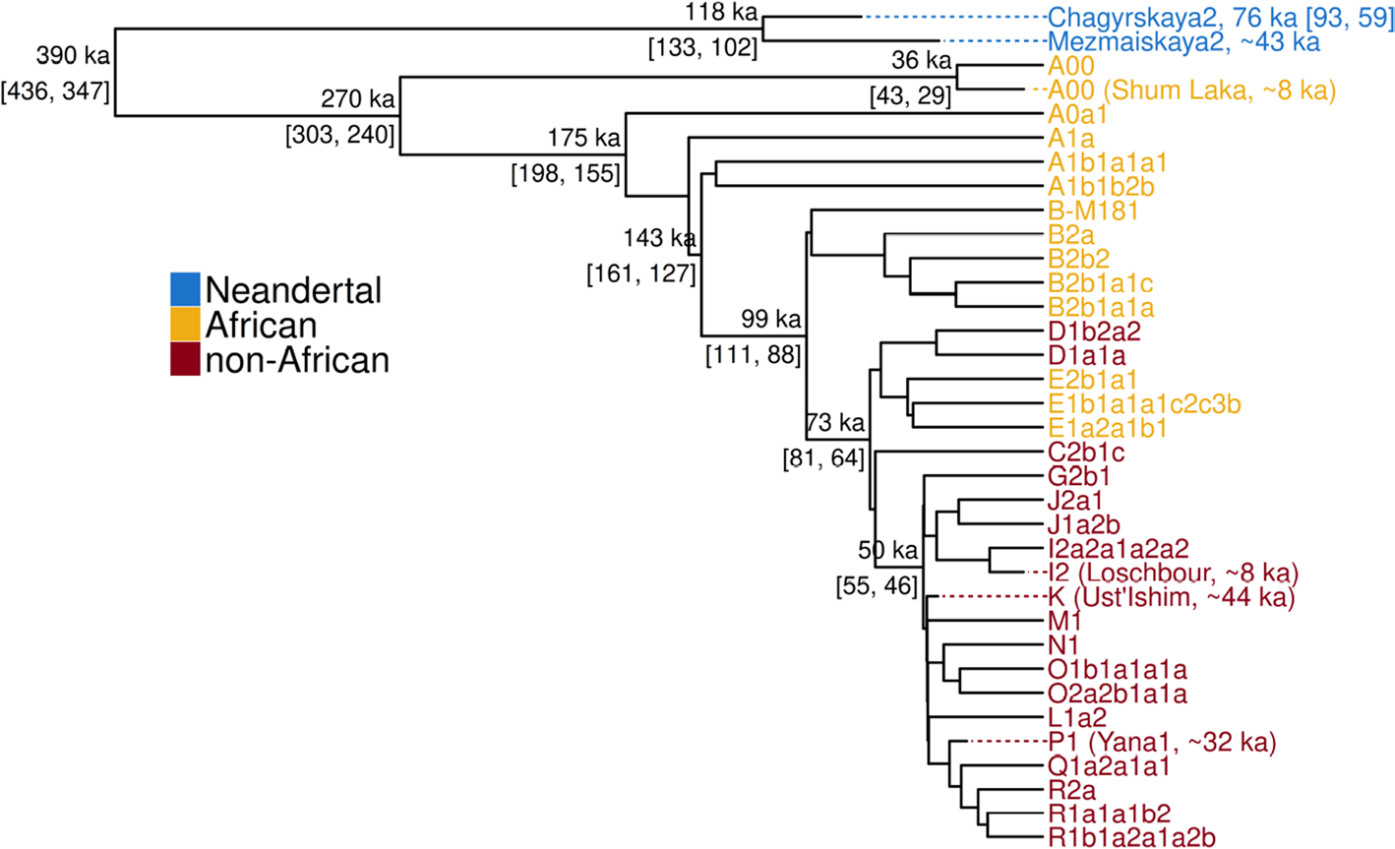
Y chromosome phylogeny reconstructed with BEAST. The TMRCA estimates, as well as the estimated age of *Chagyrskaya 2* (shown in the branch label), and their respective 95% HPD intervals are indicated on the tree. The ages of the other ancient samples were set to the estimated radiocarbon dates. The haplogroups from different populations are highlighted with different colors. The branches are to scale, in thousands of years (ka)

As we are able to calculate unbiased TMRCAs after masking divergent regions, we then used BEAST to reconstruct the Y chromosome phylogeny (see the “Materials and methods” section) based on the sites that pass the divergence filter (Fig. 6a). BEAST estimates similar TMRCAs to those estimated with the method described above, although with narrower confidence intervals. We estimated that the modern human TMRCA is 270 ka (95% HPD 303 to 240 ka) and the modern human-Neandertal TMRCA is 390 ka (95% HPD 436 to 347 ka). Our recalibrated TMRCAs are slightly older than previous estimates of 254 ka (95% CI 307 to 192 ka) for the modern human TMRCA [7] and 370 ka (95% CI 420 to 326 ka) for the modern human-Neandertal TMRCA [1], but within their confidence intervals.

**Fig. 7:**
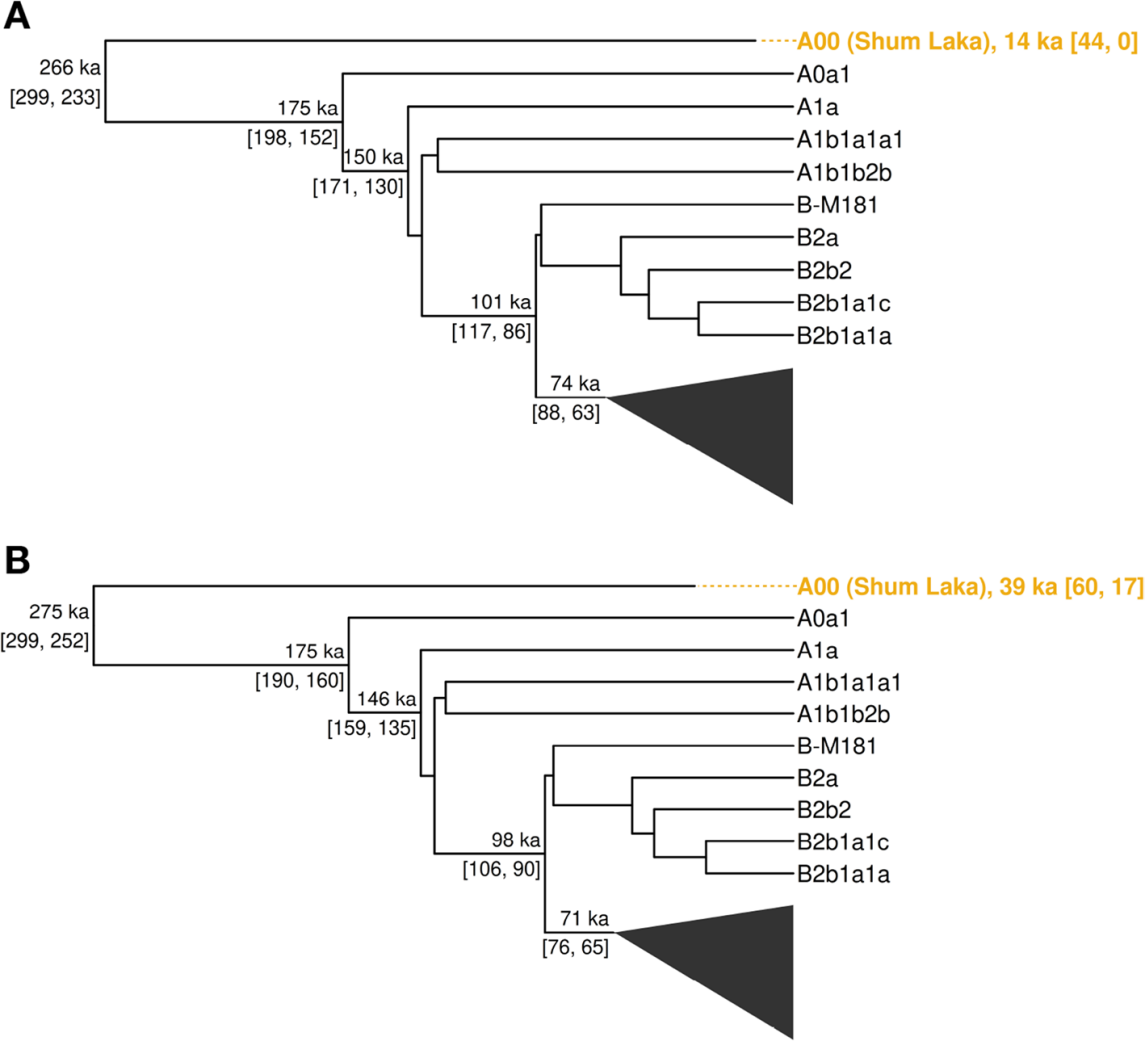
Estimating the age of the radiocarbon dated A00 individual (radiocarbon date $\sim$8 ka) using molecular dating of the Y chromosome with BEAST. Phylogenies based on the filtered (**a**) and the unfiltered (**b**) Y chromosome are shown. The branches corresponding to 22 Y chromosomes included in the analysis (Additional file 1: Table S1) were collapsed. The TMRCA estimates and their 95% HPD intervals are indicated on the tree. The age of the A00 individual, along with its associated 95% HPD interval, is indicated in the branch label

We then tested whether we can use BEAST to estimate the age of a shotgun sequenced ancient individual from Shum Laka, Cameroon dated to $\sim$8 ka (7970 to 7800 calibrated years before present) who carried the A00 Y haplogroup using just the Y chromosome. For this analysis, we set a wide prior for the age of the ancient individual (− 50,000 to 200,000) and did not include the present-day A00 individuals. Employing the same parameters used for generating the full phylogeny above, we constructed one phylogeny with the filtered Y chromosome and one with the unfiltered Y chromosome (Fig. 7). We find that using the unfiltered Y chromosome to construct the phylogeny yields an age-estimate for the ancient A00 individual that is about 30 thousand years older than the radiocarbon date due to the reference bias that leads to an underestimate of the number of mutations on this branch. If we use the filtered Y chromosome regions, we obtain an age estimate of 14 ka (95% CI 44 to 0) which is reasonably close to the radiocarbon date (albeit with wide confidence intervals) suggesting that filtering is necessary for accurate age estimation.

We also re-estimated an age for the *Chagyrskaya 2* Neandertal—previously dated to $\sim$64 ka (95% CI 83 to 48) [15]. Our estimate of 76 ka (95% CI 93 to 59) is consistent with a Neandertal occupation of Chagyrskaya Cave between 92 ka and 49 ka, and close to the genetic age of *Chagyrskaya 8* ($\sim$80 ka), another individual from the same cave, whose age was estimated from a high-coverage genome [27].

In summary, although the re-estimated TMRCAs are not significantly different from previous estimates, these analyses show that the divergence filtering is necessary to obtain Y chromosome-based age estimates for ancient individuals with Y chromosomes that are diverged from the human reference.

## Discussion

We show here that variation in Y chromosome branch lengths is at least in part explained by biases introduced by alignment to a single Y reference genome representing a single haplogroup. Although we do not observe any significant haplogroup-specific variation after accounting for the effect of reference bias (Additional file 1: Fig. S4 and Additional file 1: Fig. S5), a larger Y chromosome dataset with multiple samples representing specific lineages in populations of interest may allow for the detection of subtle branch length differences that result from changes in mutation rate or generation time.

The Y chromosome is more affected than the autosomes by this reference bias due to its highly repetitive nature and extensive structural variation. We show that restricting analyses of human Y chromosomes to regions with a human-chimpanzee divergence of $\le$1.9% allows accurate estimation of branch lengths and, therefore, unbiased estimates of the human Y chromosome phylogeny. However, doing so reduces the (already limited) amount of the Y chromosome that is accessible for analysis by more than half, leading to wider confidence intervals in both TMRCA and age estimates. For analyses of Y chromosomes in specific populations, it may be possible to recover more of the Y sequence by alignment to the closest high-quality Y chromosome assembly [19].

In contrast to using a single linear reference genome, approaches that incorporate Y chromosome haplotype diversity in the reference to which sequence reads are aligned, known as “pangenomes,” have been shown to reduce the effect of reference bias when used as a reference for short-read mapping [28, 29] and could potentially maximize the amount of the Y chromosome that is accessible for comparison. The repetitive nature of the Y chromosome, along with its extensive structural variation, could make it a particularly good target for a pangenome-based approach. A number of high-quality Y chromosome assemblies that could be used to construct a pangenome reference [19] are already available, although methods to incorporate ancient Y chromosome variation into pangenome graphs have yet to be explored and are an exciting direction for future research.

We expect our divergence-filtering approach to be particularly useful for analyses of archaic human (i.e., Neandertal and Denisovan) Y chromosomes that are substantially diverged from the human reference, and for which the construction of pangenome graphs remains challenging. These Y chromosomes will be most affected by reference bias, so methods that rely on comparing the number of private mutations on archaic Y chromosomes to those of present-day human Y chromosomes will underestimate the archaic branch length if they do not account for this bias.

## Materials and methods

### Dataset

The dataset includes high-coverage Y chromosome data from present-day and ancient modern humans as well as from Neandertals (Additional file 1: Table S1). For the present day humans, a subset of individuals from the Human Genome Diversity Project (HGDP) [9] dataset were selected to represent the Y chromosome phylogeny with a particular focus toward individuals with Y chromosomes that are highly diverged from the human reference Y chromosome. We also included two individuals (carrying Y chromosomes representing the A haplogroup) from the high-coverage resequencing of the 1000 Genomes Project (1KGP) [10].

The dataset also contains Y chromosomes from two closely related individuals with the A00 haplogroup [7]. Due to the relatively low coverage of the sequence data for these two A00 Y chromosomes, we followed the approach of the original publication and merged the two A00 Y chromosomes into a single BAM file.

To calibrate the Y chromosome phylogeny, we included high-coverage, shotgun-sequenced Y chromosome data from the following radiocarbon dated ancient modern humans: *Ust’Ishim* [11], *Yana1* [14], *Loschbour* [13] and an individual from Shum Laka with the A00 haplogroup [12].

We also included two high-coverage Neandertal Y chromosomes that were obtained by enrichment-capture sequencing: *Mezmaiskaya2* [1] and *Chagyrskaya2* [15].

### Data processing

Sequences were aligned using BWA-MEM (v0.7.17) [30] for present-day individuals and BWA-ALN (with ancient parameters—“-n 0.01 -o 2 -l 16500”) for ancient individuals. For the present-day HGDP and 1KGP individuals, adapter sequences were identified and flagged using MarkIlluminaAdapters from Picard tools (v2.18.29) before alignment. For the ancient samples, we used the merged and adapter-trimmed sequences as published (Additional file 1: Table S1).

The aligned BAM files for each individual were filtered with samtools [31] to retain reads at least 35 bp in length and with a minimum mapping quality of 25, and PCR duplicates were removed using bam-rmdup (v0.2, https://github.com/mpieva/biohazard-tools/). We also removed sequences with indels as we observed an increased frequency of genotyping errors around indels.

We restricted our analyses to uniquely mappable regions of the Y chromosome so as to minimize errors caused by ambiguous alignment of short reads. To exclude non-unique regions of the hg19 Y chromosome, we used the map35_100 filter from [32] which retains only positions where all 35mers overlapping that position do not map to any other position in the genome allowing up to one mismatch. We also applied the capture_full filter from [1], which additionally masks tandem repeats identified by Tandem Repeat Finder [33].

Genotypes were called with snpAD [34]—a genotyper that accounts for ancient DNA damage—using bases with a minimum base quality of 30. For consistency, snpAD was used to call genotypes for both ancient and modern individuals, however, position-specific error rates were estimated independently for each sample. As snpAD is designed to call diploid genotypes, heterozygous genotype calls were converted to homozygous genotypes choosing the lowest homozygous posterior probability as calculated by snpAD. Finally, following [32], genotypes were filtered based on maximum GC-corrected coverage cutoffs for each individual (see the “Availability of data and materials” section) in order to remove potential duplications or regions with spurious alignments of microbial sequences.

### Methods to estimate branch shortening

#### Branch length difference

To calculate the difference in branch length with respect to a reference individual, we counted the number of derived mutations on each branch (using the chimpanzee genome to infer the ancestral state) with the *BranchShortening.get_mutations_vs_individual* function from [35]. The UCSC whole genome alignments between the human reference genome (hg19) and the chimpanzee reference genome (panTro6) were used to determine ancestral genotypes.

#### Estimating the human Y chromosome mutation rate

A Y chromosome from an ancient human individual with an accurately estimated age can be used to determine the human Y chromosome mutation rate [36]. The *Ust’Ishim* individual is estimated, based on radiocarbon dating, to have lived around 44,000 years ago (45,930–42,904 calibrated years before present; [11]). We used the high-coverage genome sequence of *Ust’Ishim* to determine the number of derived mutations on the branch leading to *Ust’Ishim*, and compared this to an individual living today. Normalizing the number of mutations accumulated per year by the total number of positions used for the comparison results in the Y chromosome mutation rate in units of mutations per position per year.

#### Estimating the mutation rate on a shortened branch

To estimate the mutation rate that would result in a shortened branch when comparing two Y chromosome lineages, we first calculate the expected branch length difference using the known ages of the two individuals and the expected mutation rate. We then calculate the proportion of missing mutations using the observed branch length difference and adjust the expected mutation rate, based on this proportion, to estimate the mutation rate on the shorter branch.

#### Estimating TMRCAs of modern and archaic human Y chromosomes

We used the method described in [1] to estimate the TMRCAs of modern and archaic Y chromosomes. Briefly, the method first estimates the modern human TMRCA by counting the differences between a present-day African Y chromosome and a present-day non-African Y chromosome and converting this count to time using a mutation rate. The archaic TMRCA is then computed by scaling the modern human TMRCA based on the fold increase in divergence between one of the present-day Y chromosomes and the archaic Y chromosome relative to the divergence between the two present-day Y chromosomes.

### Mapping to the chimpanzee reference Y chromosome

We replaced the hg19 Y chromosome with the chimpanzee reference (panTro6) Y chromosome (hg19_panTro6_Y) and aligned all sequence reads from two ancient genomes (*Ust’Ishim* and *Shum Laka*) to this reference. To conduct a fair comparison, we downsampled the aligned sequences of *Ust’Ishim* to the same coverage distribution and read length distribution of the aligned sequences of *Shum Laka*. We then called genotypes with the same pipeline that we use to call genotypes from the sequences aligned to the human hg19 reference.

### Estimating the percentage change in mutation rate in response to a change in generation time

To estimate the relative change in mutation rate corresponding to a change in generation time, we used the relationship between paternal age (*a*) and the autosomal germline mutation rate ($\mu$, in units of male-transmitted mutations per generation) defined in [23] as

$$\begin{aligned} \mu = 6.05 + 1.51 \times a. \end{aligned}$$

We used the autosomal relationship as this relationship for the Y chromosome cannot be determined accurately due to the small size of the Y chromosome, which accumulates relatively few mutations per generation [37].

To account for the fact that a decrease in the average paternal age can result in an increase in the number of mutations over time due to an increase in the number of generations, we define the relative change in mutation rate (if the paternal age changed from $a_{current}$ to $a_{past}$) as

$$\begin{aligned} \frac{ \frac{a_{current}}{a_{past}} \mu _{past} - \mu _{current}}{\mu _{current}}. \end{aligned}$$

### Processing of long-read assembled Y chromosomes

We replaced the Y chromosome of the hg19 reference genome with the A0b Y chromosome (hg19_A0b_Y) from [19] or with the T2T Y chromosome (hg19_T2T_Y) from [18]. We then generated new map35_100 filters and ran Tandem Repeat Finder [33] on both the hg19_A0b_Y genome and the hg19_T2T_Y genome to identify the uniquely mappable regions for each genome. To call genotypes, we used the same processing pipeline as the one used for hg19.

To identify ancestral alleles, we used LASTZ [38] to align the chimpanzee reference genome (panTro6) to both the hg19_A0b_Y genome and the hg19_T2T_Y genome using the UCSC LASTZ alignment parameters for human-chimp alignments. The alignments were then chained with axtChain using the UCSC chaining parameters and the high-scoring ($> 2000$) alignments were extracted from which we called panTro6 genotypes.

### Human-chimp sequence divergence

To compute human-chimp sequence divergence for each base of the hg19 Y chromosome, we extracted the Y chromosome alignments from the UCSC hg19-panTro6 pairwise alignment file (hg19.panTro6.synNet.maf.gz) and used the *maf_to_pairwise_identity.py* script from [39] to determine the alignment state (i.e., match, mismatch, insertion, deletion, no alignment) for each position in the hg19 Y chromosome. We then used a 400-bp sliding window centered on each position to compute human-chimp sequence divergence as

$$\begin{aligned} 1 - \frac{n_{match}}{n_{total}}, \end{aligned}$$

where $n_{match}$ is the number of matching bases and $n_{total}$ is the total number of aligned bases in each window. Due to the fragmented nature of the uniquely mappable fraction of the human Y chromosome, we additionally required a minimum number of human-chimp aligned bases (200 bp) to compute the sequence divergence for a position.

### Bayesian phylogenetic analysis

We used BEAST2 (v2.6.7) [26] to reconstruct the Y chromosome phylogeny and estimate the TMRCAs. As BEAST requires a FASTA file as input, the *VcfHandler.convert_vcf_dataframe_to_fasta_file* function from [35] was used to convert the Y chromosome VCF data to FASTA format. Only variable sites were used (31,474 in total) as input to the BEAST analysis in order to reduce the computational complexity of the phylogenetic reconstruction, although we defined the nucleotide composition of the invariant sites by editing the BEAST .xml file as was done in [7] (Additional file 1: Table S7).

We used the BEAST2 bModelTest package [40] to estimate the most appropriate nucleotide substitution model for our data set. bModelTest averages over all substitution models while simultaneously reconstructing the phylogeny. To select the tree model and the clock model that best fit our dataset, we estimated the marginal likelihood for each model combination using a path sampling approach as implemented in the BEAST2 MODEL_SELECTION package (Additional file 1: Table S8).

To generate the full phylogeny using the best fitting models (the strict clock model together with the Bayesian Skyline tree model), we performed four independent MCMC runs of 55 million iterations with a 10 million iteration pre-burn-in stage, sampling every 5000 steps. We used Tracer v1.7 [41] to examine the output of each run and confirm that all ESS (Effective Sample Size) values were greater than 200. We then combined the resulting log files with logcombiner using a 10% burn-in for each run and generated a single best supported tree with TreeAnnotator. ESS values from the combined log file are shown in Additional file 1: Table S9.

## Supplementary Information

Additional file 1: Supplementary materials including supplementary figures and tables.

## Data Availability

All sequence data used are publicly available (Additional file 1: Table S1, [42–50]). The merged genotype files and filters used for the analysis are available from Zenodo (https://doi.org/10.5281/zenodo.13939592) [51]. All code is available, under the GPL-3.0 license, from Github (https://github.com/yanivsw/y_chr_reference_bias) [35] and Zenodo (https://doi.org/10.5281/zenodo.13970734) [52].

## References

[1] Petr M, Hajdinjak M, Fu Q, Essel E, Rougier H, Crevecoeur I, et al. The Evolutionary History of Neanderthal and Denisovan Y Chromosomes. Science. 2020;369(6511):1653–6. 10.1126/science.abb6460. 32973032 10.1126/science.abb6460

[2] Wei W, Ayub Q, Chen Y, McCarthy S, Hou Y, Carbone I, et al. A Calibrated Human Y-chromosomal Phylogeny Based on Resequencing. Genome Res. 2013;23(2):388–95. 10.1101/gr.143198.112. 23038768 10.1101/gr.143198.112 PMC3561879

[3] Scozzari R, Massaia A, Trombetta B, Bellusci G, Myres NM, Novelletto A, et al. An Unbiased Resource of Novel SNP Markers Provides a New Chronology for the Human Y Chromosome and Reveals a Deep Phylogenetic Structure in Africa. Genome Res. 2014;24(3):535–44. 10.1101/gr.160788.113. 24395829 10.1101/gr.160788.113 PMC3941117

[4] Hallast P, Batini C, Zadik D, Maisano Delser P, Wetton JH, Arroyo-Pardo E, et al. The Y-Chromosome Tree Bursts into Leaf: 13,000 High-Confidence SNPs Covering the Majority of Known Clades. Mol Biol Evol. 2015;32(3):661–73. 10.1093/molbev/msu327. 25468874 10.1093/molbev/msu327 PMC4327154

[5] Barbieri C, Hübner A, Macholdt E, Ni S, Lippold S, Schröder R, et al. Refining the Y Chromosome Phylogeny with Southern African Sequences. Hum Genet. 2016;135(5):541–53. 10.1007/s00439-016-1651-0. 27043341 10.1007/s00439-016-1651-0 PMC4835522

[6] Naidoo T, Xu J, Vicente M, Malmström H, Soodyall H, Jakobsson M, et al. Y-Chromosome Variation in Southern African Khoe-San Populations Based on Whole-Genome Sequences. Genome Biol Evol. 2020;12(7):1031–9. 10.1093/gbe/evaa098. 32697300 10.1093/gbe/evaa098 PMC7375190

[7] Karmin M, Saag L, Vicente M, Sayres MAW, Järve M, Talas UG, et al. A Recent Bottleneck of Y Chromosome Diversity Coincides with a Global Change in Culture. Genome Res. 2015;25(4):459–66. 10.1101/gr.186684.114. 25770088 10.1101/gr.186684.114 PMC4381518

[8] Mendez FL, Poznik GD, Castellano S, Bustamante CD. The Divergence of Neandertal and Modern Human Y Chromosomes. Am J Hum Genet. 2016;98(4):728–34. 10.1016/j.ajhg.2016.02.023. 27058445 10.1016/j.ajhg.2016.02.023 PMC4833433

[9] Bergström A, McCarthy SA, Hui R, Almarri MA, Ayub Q, Danecek P, et al. Insights into Human Genetic Variation and Population History from 929 Diverse Genomes. Science. 2020;367(6484):eaay5012. 10.1126/science.aay5012. 32193295 10.1126/science.aay5012 PMC7115999

[10] Byrska-Bishop M, Evani US, Zhao X, Basile AO, Abel HJ, Regier AA, et al. High-Coverage Whole-Genome Sequencing of the Expanded 1000 Genomes Project Cohort Including 602 Trios. Cell. 2022;185(18):3426-3440.e19. 10.1016/j.cell.2022.08.004. 36055201 10.1016/j.cell.2022.08.004 PMC9439720

[11] Fu Q, Li H, Moorjani P, Jay F, Slepchenko SM, Bondarev AA, et al. Genome Sequence of a 45,000-Year-Old Modern Human from Western Siberia. Nature. 2014;514(7523):445–9. 10.1038/nature13810. 25341783 10.1038/nature13810 PMC4753769

[12] Lipson M, Ribot I, Mallick S, Rohland N, Olalde IN, Adamski N, et al. Ancient West African Foragers in the Context of African Population History. Nature. 2020;577(7792):665–70. 10.1038/s41586-020-1929-1. 31969706 10.1038/s41586-020-1929-1 PMC8386425

[13] Lazaridis I, Patterson N, Mittnik A, Renaud G, Mallick S, Kirsanow K, et al. Ancient Human Genomes Suggest Three Ancestral Populations for Present-Day Europeans. Nature. 2014;513(7518):409–13. 10.1038/nature13673. 25230663 10.1038/nature13673 PMC4170574

[14] Sikora M, Pitulko VV, Sousa VC, Allentoft ME, Vinner L, Rasmussen S, et al. The Population History of Northeastern Siberia since the Pleistocene. Nature. 2019;570(7760):182–8. 10.1038/s41586-019-1279-z. 31168093 10.1038/s41586-019-1279-z PMC7617447

[15] Skov L, Peyrégne S, Popli D, Iasi LNM, Devièse T, Slon V, et al. Genetic Insights into the Social Organization of Neanderthals. Nature. 2022;610(7932):519–25. 10.1038/s41586-022-05283-y. 36261548 10.1038/s41586-022-05283-y PMC9581778

[16] Günther T, Nettelblad C. The Presence and Impact of Reference Bias on Population Genomic Studies of Prehistoric Human Populations. PLoS Genet. 2019;15(7):e1008302. 10.1371/journal.pgen.1008302. 31348818 10.1371/journal.pgen.1008302 PMC6685638

[17] Peyrégne S, Slon V, Mafessoni F, De Filippo C, Hajdinjak M, Nagel S, et al. Nuclear DNA from Two Early Neandertals Reveals 80,000 Years of Genetic Continuity in Europe. Sci Adv. 2019;5(6):eaaw5873. 10.1126/sciadv.aaw5873. 31249872 10.1126/sciadv.aaw5873 PMC6594762

[18] Rhie A, Nurk S, Cechova M, Hoyt SJ, Taylor DJ, Altemose N, et al. The Complete Sequence of a Human Y Chromosome. Nature. 2023. 10.1038/s41586-023-06457-y. 10.1038/s41586-023-06457-y PMC10752217 37612512

[19] Hallast P, Ebert P, Loftus M, Yilmaz F, Audano PA, Logsdon GA, et al. Assembly of 43 Human Y Chromosomes Reveals Extensive Complexity and Variation. Nature. 2023. 10.1038/s41586-023-06425-6. 10.1038/s41586-023-06425-6 PMC10726138 37612510

[20] Skov L, The Danish Pan Genome Consortium, Schierup MH. Analysis of 62 Hybrid Assembled Human Y Chromosomes Exposes Rapid Structural Changes and High Rates of Gene Conversion. PLOS Genet. 2017;13(8):e1006834. 10.1371/journal.pgen.1006834. 10.1371/journal.pgen.1006834 PMC5591018 28846694

[21] Makova KD, Pickett BD, Harris RS, Hartley GA, Cechova M, Pal K, et al. The Complete Sequence and Comparative Analysis of Ape Sex Chromosomes. Nature. 2024;630(8016):401–11. 10.1038/s41586-024-07473-2. 38811727 10.1038/s41586-024-07473-2 PMC11168930

[22] Fenner JN. Cross-Cultural Estimation of the Human Generation Interval for Use in Genetics-Based Population Divergence Studies. Am J Phys Anthropol. 2005;128(2):415–23. 10.1002/ajpa.20188. 15795887 10.1002/ajpa.20188

[23] Jónsson H, Sulem P, Kehr B, Kristmundsdottir S, Zink F, Hjartarson E, et al. Parental Influence on Human Germline de Novo Mutations in 1,548 Trios from Iceland. Nature. 2017;549(7673):519–22. 10.1038/nature24018. 28959963 10.1038/nature24018

[24] Sun C, Skaletsky H, Birren B, Devon K, Tang Z, Silber S, et al. An azoospermic man with a de novo point mutation in the Y-chromosomal gene USP9Y. Nat Genet. 1999;23(4):429–32. 10.1038/70539. 10581029 10.1038/70539

[25] Skaletsky H, Kuroda-Kawaguchi T, Minx PJ, Cordum HS, Hillier L, Brown LG, et al. The male-specific region of the human Y chromosome is a mosaic of discrete sequence classes. Nature. 2003;423(6942):825–37. 10.1038/nature01722. 12815422 10.1038/nature01722

[26] Bouckaert R, Vaughan TG, Barido-Sottani J, Duchêne S, Fourment M, Gavryushkina A, et al. BEAST 2.5: An Advanced Software Platform for Bayesian Evolutionary Analysis. PLOS Comput Biol. 2019;15(4):e1006650. 10.1371/journal.pcbi.1006650. 30958812 10.1371/journal.pcbi.1006650 PMC6472827

[27] Mafessoni F, Grote S, De Filippo C, Slon V, Kolobova KA, Viola B, et al. A High-Coverage Neandertal Genome from Chagyrskaya Cave. Proc Natl Acad Sci. 2020;117(26):15132–6. 10.1073/pnas.2004944117. 32546518 10.1073/pnas.2004944117 PMC7334501

[28] Liao WW, Asri M, Ebler J, Doerr D, Haukness M, Hickey G, et al. A Draft Human Pangenome Reference. Nature. 2023;617(7960):312–24. 10.1038/s41586-023-05896-x. 37165242 10.1038/s41586-023-05896-x PMC10172123

[29] Martiniano R, Garrison E, Jones ER, Manica A, Durbin R. Removing Reference Bias and Improving Indel Calling in Ancient DNA Data Analysis by Mapping to a Sequence Variation Graph. Genome Biol. 2020;21(1):250. 10.1186/s13059-020-02160-7. 32943086 10.1186/s13059-020-02160-7 PMC7499850

[30] Li H, Durbin R. Fast and Accurate Long-Read Alignment with Burrows-Wheeler Transform. Bioinformatics. 2010;26(5):589–95. 10.1093/bioinformatics/btp698. 20080505 10.1093/bioinformatics/btp698 PMC2828108

[31] Danecek P, Bonfield JK, Liddle J, Marshall J, Ohan V, Pollard MO, et al. Twelve Years of SAMtools and BCFtools. GigaScience. 2021;10(2):giab008. 10.1093/gigascience/giab008. 33590861 10.1093/gigascience/giab008 PMC7931819

[32] Prüfer K, Racimo F, Patterson N, Jay F, Sankararaman S, Sawyer S, et al. The Complete Genome Sequence of a Neanderthal from the Altai Mountains. Nature. 2014;505(7481):43–9. 10.1038/nature12886. 24352235 10.1038/nature12886 PMC4031459

[33] Benson G. Tandem Repeats Finder: A Program to Analyze DNA Sequences. Nucleic Acids Res. 1999;27(2):573–80. 10.1093/nar/27.2.573. 9862982 10.1093/nar/27.2.573 PMC148217

[34] Prüfer K. snpAD: An Ancient DNA Genotype Caller. Bioinformatics. 2018;34(24):4165–71. 10.1093/bioinformatics/bty507. 29931305 10.1093/bioinformatics/bty507 PMC6289138

[35] Swiel Y, Kelso J, Peyrégne S. Resolving the source of branch length variation in the Y chromosome phylogeny. Github. 2024. https://github.com/yanivsw/y_chr_reference_bias. Accessed 12 Dec 2024. 10.1186/s13059-024-03468-4 PMC11702058 39762943

[36] Balanovsky O. Toward a Consensus on SNP and STR Mutation Rates on the Human Y-chromosome. Hum Genet. 2017;136(5):575–90. 10.1007/s00439-017-1805-8. 28455625 10.1007/s00439-017-1805-8

[37] Helgason A, Einarsson AW, et al. The Y-chromosome Point Mutation Rate in Humans. Nat Genet. 2015;47(5):453–7. 10.1038/ng.3171. 25807285 10.1038/ng.3171

[38] Harris RS. Improved Pairwise Alignment of Genomic DNA PhD thesis (United States – Pennsylvania). 84 pp. isbn: 9780549431701.

[39] Cechova M, Vegesna R, Tomaszkiewicz M, Harris RS, Chen D, Rangavittal S, et al. Dynamic Evolution of Great Ape Y Chromosomes. Proc Natl Acad Sci. 2020;117(42):26273–80. 10.1073/pnas.2001749117. 33020265 10.1073/pnas.2001749117 PMC7585023

[40] Bouckaert RR, Drummond AJ. bModelTest: Bayesian Phylogenetic Site Model Averaging and Model Comparison. BMC Evol Biol. 2017;17(1):42. 10.1186/s12862-017-0890-6. 28166715 10.1186/s12862-017-0890-6 PMC5294809

[41] Rambaut A, Drummond AJ, Xie D, Baele G, Suchard MA. Posterior Summarization in Bayesian Phylogenetics Using Tracer 1.7. Syst Biol. 2018;67(5):901–4. 10.1093/sysbio/syy032. 29718447 10.1093/sysbio/syy032 PMC6101584

[42] Bergström A, McCarthy SA, Hui R, Almarri MA, Ayub Q, Danecek P, et al. Insights into human genetic variation and population history from 929 diverse genomes. Datasets. European Nucleotide Archive. 2016. https://www.ebi.ac.uk/ena/browser/view/PRJEB6463. Accessed 12 Dec 2024. 10.1126/science.aay5012 PMC7115999 32193295

[43] Byrska-Bishop M, Evani US, Zhao X, Basile AO, Abel HJ, Regier AA, et al. High-coverage whole-genome sequencing of the expanded 1000 Genomes Project cohort including 602 trios. Datasets. European Nucleotide Archive. 2020. https://www.ebi.ac.uk/ena/browser/view/PRJEB37677. Accessed 12 Dec 2024. 10.1016/j.cell.2022.08.004 PMC9439720 36055201

[44] Karmin M, Saag L, Vicente M, Sayres MAW, Järve M, Talas UG, et al. A recent bottleneck of Y chromosome diversity coincides with a global change in culture. Datasets. Estonian Biocentre. 2016. https://evolbio.ut.ee/chrY. Accessed 12 Dec 2024.

[45] Lazaridis I, Patterson N, Mittnik A, Renaud G, Mallick S, Kirsanow K, et al. Ancient human genomes suggest three ancestral populations for present-day Europeans. Datasets. Eur Nucleotide Arch. 2016. https://www.ebi.ac.uk/ena/browser/view/PRJEB6272. Accessed 12 Dec 2024. 10.1038/nature13673 PMC4170574 25230663

[46] Fu Q, Li H, Moorjani P, Jay F, Slepchenko SM, Bondarev AA, et al. Genome sequence of a 45,000-year-old modern human from western Siberia. Datasets. Eur Nucleotide Arch. 2016. https://www.ebi.ac.uk/ena/browser/view/PRJEB6622. Accessed 12 Dec 2024. 10.1038/nature13810 PMC4753769 25341783

[47] Sikora M, Pitulko VV, Sousa VC, Allentoft ME, Vinner L, Rasmussen S, et al. The population history of northeastern Siberia since the Pleistocene. Datasets. Eur Nucleotide Arch. 2018. https://www.ebi.ac.uk/ena/browser/view/PRJEB29700. Accessed 12 Dec 2024.

[48] Lipson M, Ribot I, Mallick S, Rohland N, Olalde I, Adamski N, et al. Ancient West African foragers in the context of African population history. Datasets. Eur Nucleotide Arch. 2020. https://www.ebi.ac.uk/ena/browser/view/PRJEB32086. Accessed 12 Dec 2024. 10.1038/s41586-020-1929-1 PMC8386425 31969706

[49] Petr M, Hajdinjak M, Fu Q, Essel E, Rougier H, Crevecoeur I, et al. The evolutionary history of Neanderthal and Denisovan Y chromosomes. Datasets. Eur Nucleotide Arch. 2020. https://www.ebi.ac.uk/ena/browser/view/PRJEB39390. Accessed 12 Dec 2024. 10.1126/science.abb6460 32973032

[50] Skov L, Peyrégne S, Popli D, Iasi LNM, Devièse T, Slon V, et al. Genetic insights into the social organization of Neanderthals. Datasets. Eur Nucleotide Arch. 2022. https://www.ebi.ac.uk/ena/browser/view/PRJEB55327. Accessed 12 Dec 2024. 10.1038/s41586-022-05283-y PMC9581778 36261548

[51] Swiel Y, Kelso J, Peyrégne S. Resolving the source of branch length variation in the Y chromosome phylogeny - Y chromosome dataset. Zenodo. 2024. 10.5281/ZENODO.12635539. 10.1186/s13059-024-03468-4 PMC11702058 39762943

[52] Swiel Y, Kelso J, Peyrégne S. Resolving the source of branch length variation in the Y chromosome phylogeny - Jupyter notebooks and code. Zenodo. 2024. 10.5281/ZENODO.13970733. 10.1186/s13059-024-03468-4 PMC11702058 39762943

