# Supplementary material for: Resolving the source of branch length variation in the Y chromosome phylogeny
Source: Genome Biol. 2025 Jan 6;26:4. doi: 10.1186/s13059-024-03468-4 (PMC11702058; doi:10.1186/s13059-024-03468-4)
Supplement: Supplementary file 1 — Additional file 1: Supplementary materials including supplementary figures and tables. [file 13059_2024_3468_MOESM1_ESM.pdf]

Resolving the source of branch length variation in the Y chromosome phylogeny

Supplementary Materials

Bayesian Phylogenetic Analysis

The tip dates of modern samples were set to zero, while uniform priors were used to represent the ages of the ancient samples. For the radiocarbon dated ancient samples, the range of the prior was set as the 95% confidence interval based on the IntCal20 calibration curve and for *Chagyrskaya2* (who cannot be radiocarbon dated, but has been genetically dated to 64 ka [15]) we set a wide uniform prior ranging from 120 ka to 40 ka. We set the initial Y chromosome mutation rate to 7.34×10^{-10} mutations/bp/year as calculated in [1]. In order to allow BEAST to effectively estimate the Y chromosome mutation rate, we set a wide uniform prior of 4×10^{-10} to 10×10^{-10}.

The modern humans and the Neandertals were constrained as two different monophyletic groups in order to estimate their respective Y chromosome TMRCAs, and we estimated the modern human-Neandertal TMRCA by creating a third monophyletic group with only the chimpanzee as an outgroup.

To select the tree model and the clock model that best fit our dataset, we estimated the marginal likelihood for each model combination using a path sampling approach as implemented in the BEAST2 MODEL_SELECTION package. We tested the following model combinations:

- Strict clock, constant population size
- Relaxed log-normal clock, constant population size
- Strict clock, Coalescent Bayesian Skyline
- Relaxed log-normal clock, Coalescent Bayesian Skyline

For each model combination we used 50 path steps, an alpha parameter of 0.3 for the Beta distribution used to space out the steps, and a chain length of 10 million MCMC iterations for each step. The pre-burn-in stage was set at 5 million iterations and an 80% burn-in was used for each chain.

The two best supported model combinations were those with the Coalescent Bayesian Skyline tree model with slightly higher support for the relaxed clock model (Table S8). The support for the relaxed clock model over the strict clock model was not significant however (Bayes factor = 0.34), so we used the simpler model of the strict clock together with the Bayesian Skyline tree model to infer the various TMRCAs.

The nucleotide substitution model with the highest posterior probability as estimated by BEAST using the bModelTest package is the unnamed 123421 model. The nucleotide substitution rates are summarised in Table S10.

Supplementary Figures

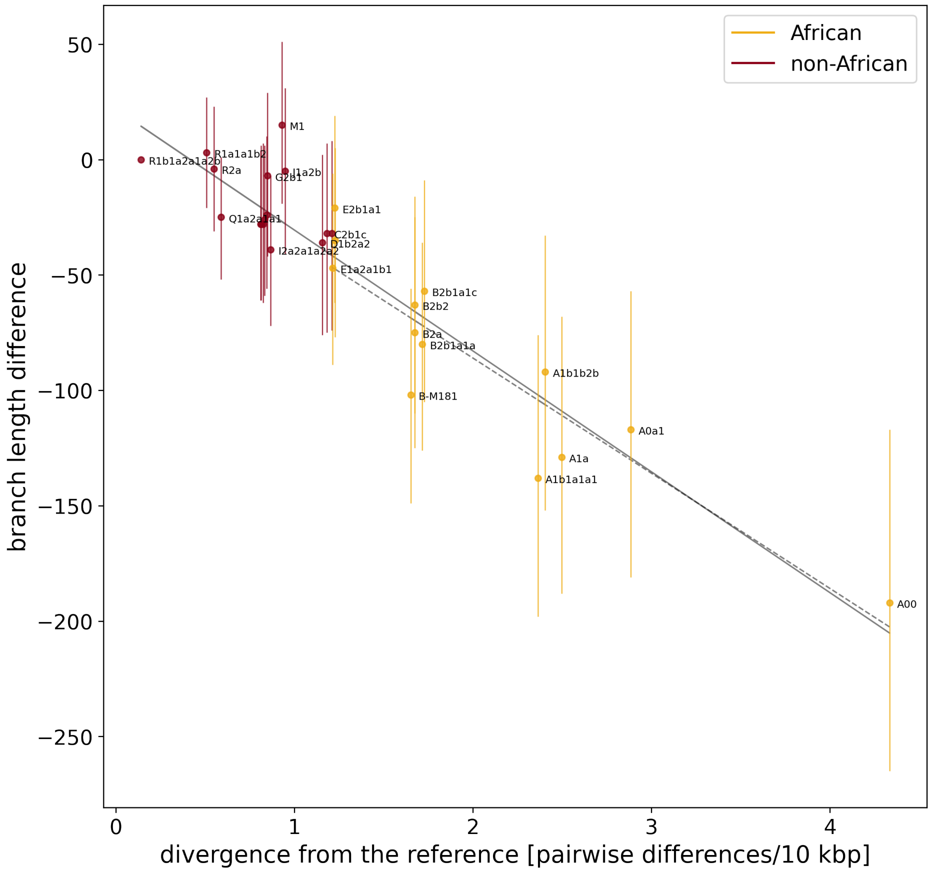

Fig. S1: Branch length differences versus divergence from the reference for all present-day Y chromosomes. The solid black line represents the straight line that best fits the data ($r = -0.936$, $P = 8.586 \times 10^{-14}$) with slope -52.39 mutations per pairwise differences per 10 kbp (kilobase pairs). The dashed black line represents the straight line fitted to the data from only the African Y chromosomes ($r = -0.916$, $P = 1.072 \times 10^{-5}$).

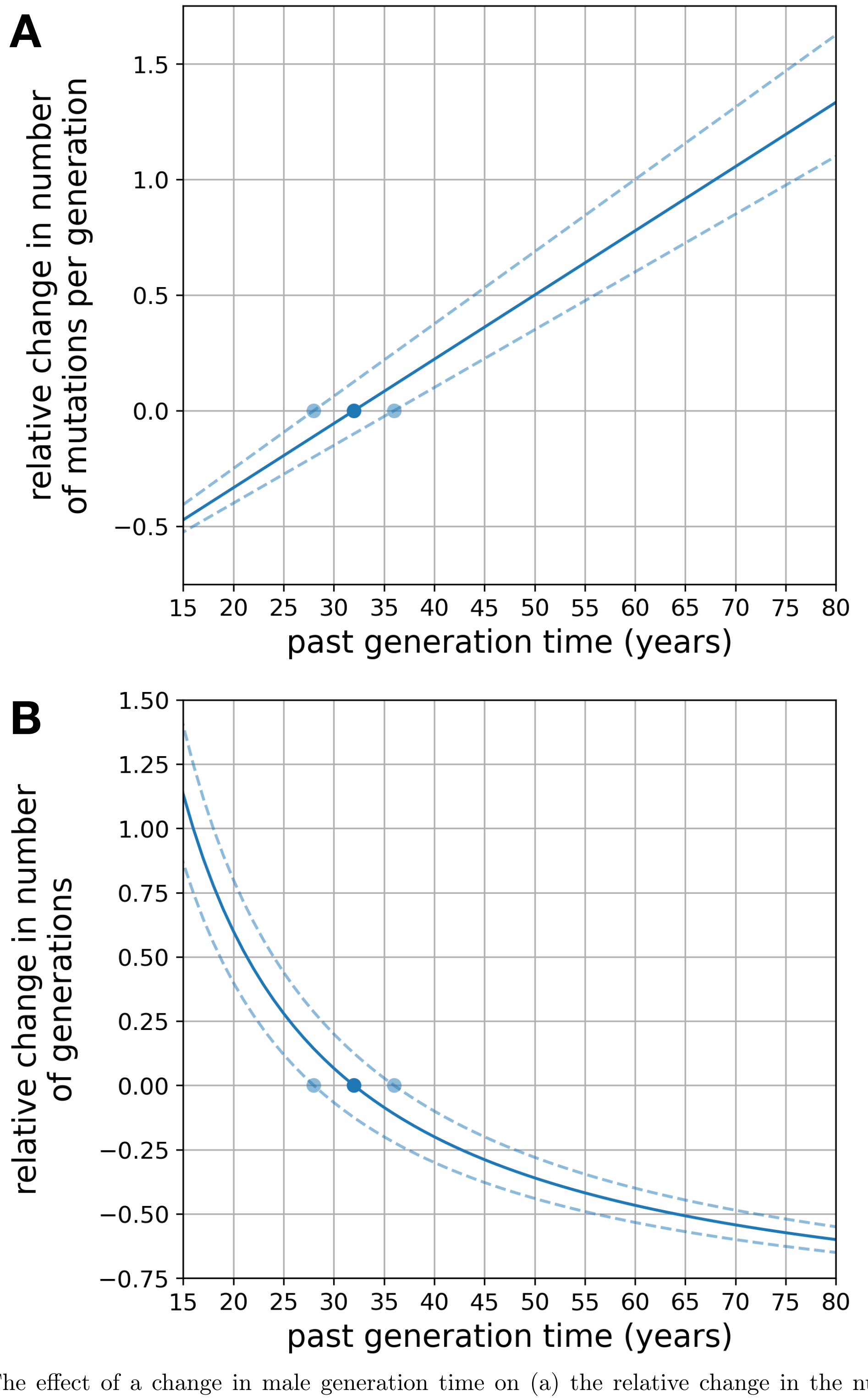

Fig. S2: The effect of a change in male generation time on (a) the relative change in the number of mutations per generation and (b) the relative change in the number of generations as a function of the past male generation time (x-axis) compared to a current value of 32 years. The dashed lines correspond to the relative change for a current generation time of 28 or 36 years.

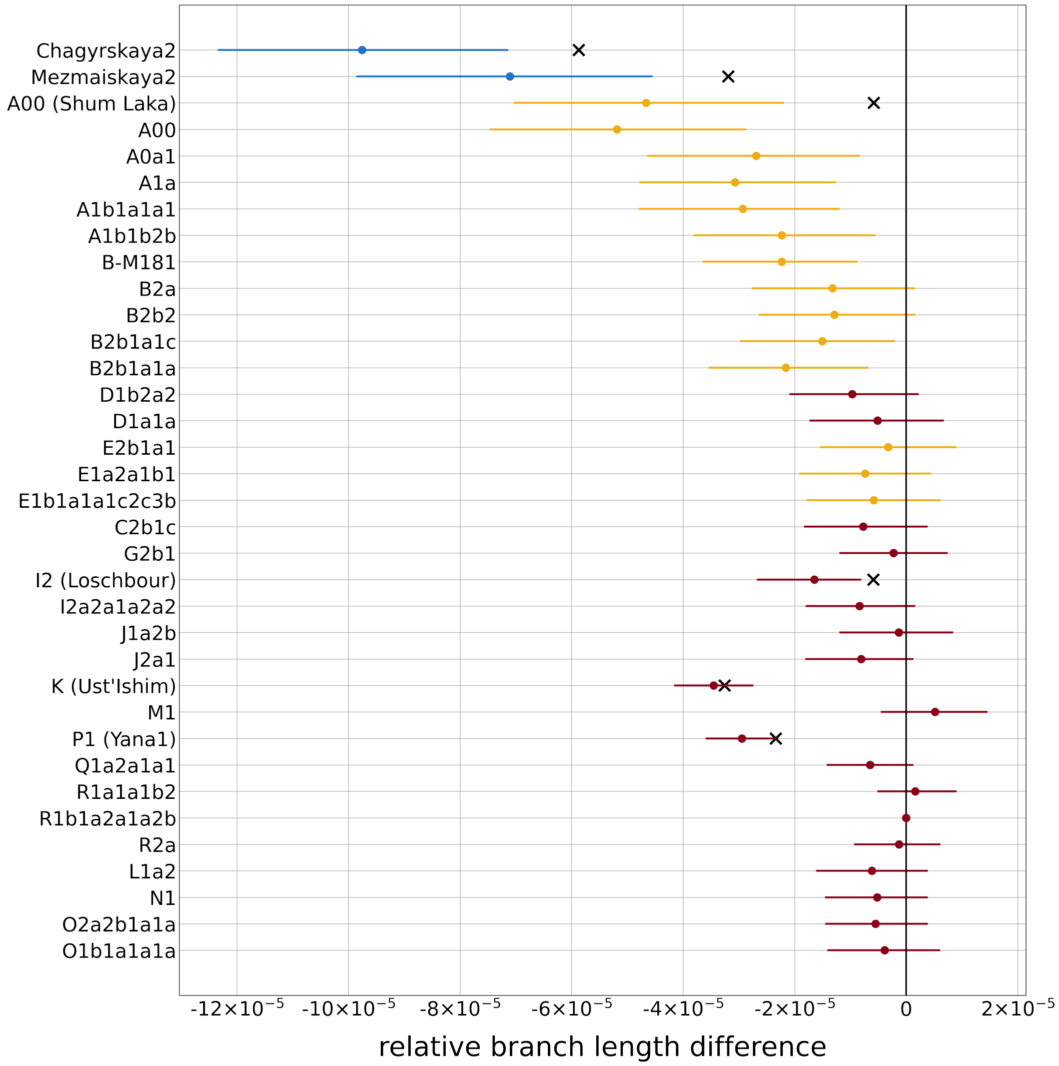

Fig. S3: Relative branch length differences compared to a present-day non-African Y chromosome (R1b1a2a1a2b) based on the X-degenerate regions of the Y chromosome.

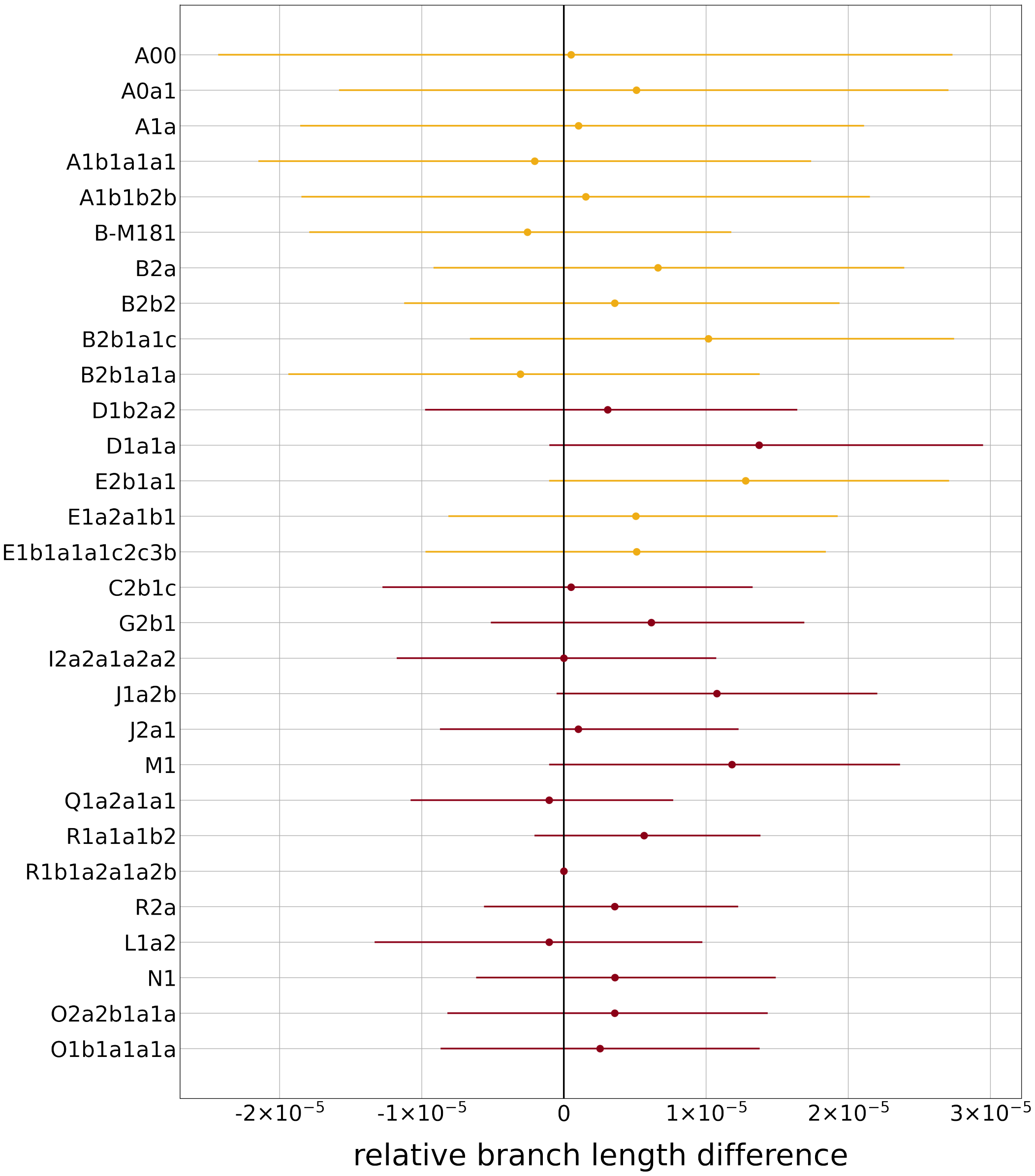

Fig. S4: Relative branch length differences compared to a present-day non-African Y chromosome (R1b1a2a1a2b) for all present-day Y chromosomes after minimising the effect of reference bias.

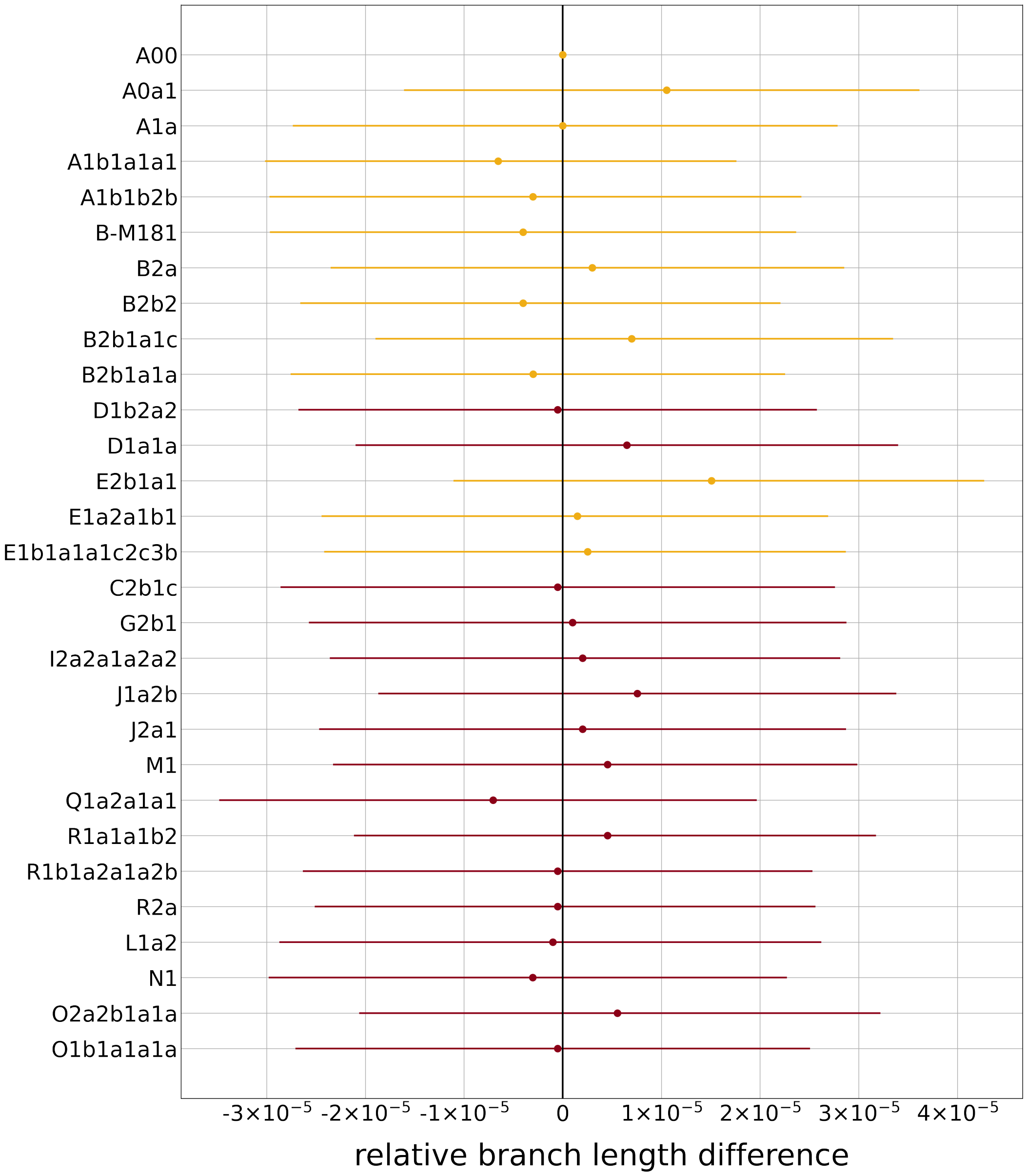

Fig. S5: Relative branch length differences compared to A00 for all present-day Y chromosomes after minimising the effect of reference bias.

Supplementary Tables

Table S1: Dataset

Individual	Data source	Sample accession	Study accession	Haplogroup	Y chromosome coverage
Chagyrskaya2	[15, 50]	SAMEA110640443	PRJEB55327	Neandertal	46.8
Mezmaiskaya2	[1, 49]	SAMEA7062760	PRJEB39390	Neandertal	15.8
Shum Laka	[12, 48]	SAMEA6104836	PRJEB32086	A00	8.5
Loschbour	[13, 45]	SAMEA2697124	PRJEB6272	I2	10.5
Ust'Ishim	[11, 46]	SAMEA2656891	PRJEB6622	K	20.0
Yana1	[14, 47]	SAMEA5684442	PRJEB29700	P1	13.8
A00	[7, 44]	GRC13292545<br>GRC13292546	PRJEB8108	A00	17.2
HG02982	[10, 43]	SAMN01761225	PRJEB37677	A0a1	14.3
HG02666	[10, 43]	SAMN00779975	PRJEB37677	A1a	15.0
HGDP01029	[9, 42]	SAMEA2580841	PRJEB6463	A1b1a1a1	15.2
HGDP01406	[9, 42]	SAMEA2580853	PRJEB6463	A1b1b2b	14.4
HGDP00931	[9, 42]	SAMEA2580871	PRJEB6463	B-M181	15.8
HGDP00478	[9, 42]	SAMEA2580834	PRJEB6463	B2a	16.5
HGDP00453	[9, 42]	SAMEA2580803	PRJEB6463	B2b2	15.0
HGDP00475	[9, 42]	SAMEA2580818	PRJEB6463	B2b1a1c	15.2
HGDP00984	[9, 42]	SAMEA2580836	PRJEB6463	B2b1a1a	16.5
HGDP00757	[9, 42]	SAMEA2581095	PRJEB6463	D1b2a2	15.9
HGDP01214	[9, 42]	SAMEA2581057	PRJEB6463	D1a1a	14.9
HGDP01031	[9, 42]	SAMEA2580848	PRJEB6463	E2b1a1	14.7
HGDP01200	[9, 42]	SAMEA2580900	PRJEB6463	E1a2a1b1	14.9
HGDP00908	[9, 42]	SAMEA2580889	PRJEB6463	E1b1a1a1c2c3b	14.7
HGDP00103	[9, 42]	SAMEA2581152	PRJEB6463	C2b1c	14.8
HGDP00213	[9, 42]	SAMEA2581196	PRJEB6463	G2b1	15.2
HGDP00127	[9, 42]	SAMEA2581169	PRJEB6463	I2a2a1a2a2	15.2
HGDP00057	[9, 42]	SAMEA2581295	PRJEB6463	J1a2b	13.5
HGDP00056	[9, 42]	SAMEA2581294	PRJEB6463	J2a1	13.7
HGDP00549	[9, 42]	SAMEA2581681	PRJEB6463	M1	14.0
HGDP01009	[9, 42]	SAMEA2581745	PRJEB6463	Q1a2a1a1	16.4
HGDP00136	[9, 42]	SAMEA2581321	PRJEB6463	R1a1a1b2	13.6
HGDP01075	[9, 42]	SAMEA2581494	PRJEB6463	R1b1a2a1a2b	17.7
HGDP00218	[9, 42]	SAMEA2581199	PRJEB6463	R2a	15.4
HGDP01298	[9, 42]	SAMEA2581140	PRJEB6463	L1a2	14.5
HGDP01192	[9, 42]	SAMEA2581001	PRJEB6463	N1	14.1
HGDP01225	[9, 42]	SAMEA2581048	PRJEB6463	O2a2b1a1a	13.9
HGDP01190	[9, 42]	SAMEA2580999	PRJEB6463	O1b1a1a1a	15.5

Table S2: The mutation rates required to explain the observed differences in branch length.

Individual 1	Individual 2	Initial mutation rate [/bp/year]	Branch length difference [%]	Adjusted mutation rate [/bp/year]
R1b1a2a1a2b	B-M181	7.34×10^{-10}	30.00	5.14×10^{-10}
B-M181	A0a1	5.14×10^{-10}	7.49	4.75×10^{-10}
A0a1	A00	4.75×10^{-10}	9.28	4.31×10^{-10}
A00	Mezmaiskaya2	4.31×10^{-10}	7.49	3.99×10^{-10}

Table S3: Results of mapping to the chimpanzee Y chromosome.

Individual	Covered positions	Mean coverage	Heterozygotes	Private derived mutations
Shum Laka	3,272,603	7.79	1,301	653
Ust'Ishim	3,474,231	7.45	1,029	700

Table S4: Human-chimp sequence divergence across the human genome.

Chromosome	Size [bp]	Aligned [%]	Divergence [%]
1	249,250,621	88.2659	1.5645
2	243,199,373	95.8074	1.6015
3	198,022,430	96.9422	1.5767
4	191,154,276	96.2699	1.6672
5	180,915,260	96.0654	1.5985
6	171,115,067	96.2549	1.5832
7	159,138,663	95.0063	1.6601
8	146,364,022	94.9971	1.7244
9	141,213,431	81.8126	1.7097
10	135,534,747	94.5770	1.6335
11	135,006,516	94.4813	1.6236
12	133,851,895	95.4340	1.5911
13	115,169,878	81.6603	1.6339
14	107,349,540	80.3624	1.6059
15	102,531,392	77.8369	1.6721
16	90,354,753	84.7200	1.8615
17	81,195,210	92.8842	1.6332
18	78,077,248	94.1312	1.6384
19	59,128,983	89.6845	1.9681
20	63,025,520	92.6997	1.6554
21	48,129,895	70.5032	1.8762
22	51,304,566	65.5224	1.9033
X	155,270,560	93.3434	1.3168
Y	59,373,566	36.1659	3.3961

Table S5: Comparing the proportion of snpAD-called heterozygotes (likely mapping errors) for the uniquely mappable part of the Y chromosome and for the uniquely mappable, divergence-filtered Y chromosome (based on a human-chimp sequence divergence cutoff of 1.9%).

Individual	Unfiltered Y chromosome		Filtered Y chromosome		Change [%]
	Heterozygotes	Proportion	Heterozygotes	Proportion	
Chagyrskaya2	85	2.00×10^{-5}	14	6.85×10^{-6}	-65.79
Mezmaiskaya2	140	3.34×10^{-5}	21	1.05×10^{-5}	-68.66
Shum Laka	159	3.96×10^{-5}	56	2.92×10^{-5}	-26.25
Loschbour	118	2.74×10^{-5}	30	1.45×10^{-5}	-46.94
Ust'Ishim	106	2.46×10^{-5}	23	1.11×10^{-5}	-54.74
Yana1	188	4.38×10^{-5}	20	9.71×10^{-6}	-77.82
A00	387	8.87×10^{-5}	44	2.09×10^{-5}	-76.39
HG02982	95	2.19×10^{-5}	15	7.19×10^{-6}	-67.18
HG02666	67	1.56×10^{-5}	4	1.94×10^{-6}	-87.58
HGDP01029	177	4.10×10^{-5}	66	3.18×10^{-5}	-22.50
HGDP01406	178	4.14×10^{-5}	80	3.86×10^{-5}	-6.92
HGDP00931	93	2.16×10^{-5}	10	4.81×10^{-6}	-77.74
HGDP00478	90	2.08×10^{-5}	8	3.83×10^{-6}	-81.61
HGDP00453	93	2.16×10^{-5}	8	3.84×10^{-6}	-82.20
HGDP00475	92	2.12×10^{-5}	6	2.87×10^{-6}	-86.48
HGDP00984	74	1.72×10^{-5}	15	7.19×10^{-6}	-58.10
HGDP00757	57	1.32×10^{-5}	7	3.38×10^{-6}	-74.41
HGDP01214	40	9.23×10^{-6}	5	2.39×10^{-6}	-74.09
HGDP01031	78	1.81×10^{-5}	13	6.25×10^{-6}	-65.41
HGDP01200	90	2.08×10^{-5}	9	4.29×10^{-6}	-79.37
HGDP00908	99	2.31×10^{-5}	7	3.37×10^{-6}	-85.40
HGDP00103	48	1.11×10^{-5}	8	3.84×10^{-6}	-65.40
HGDP00213	82	1.91×10^{-5}	3	1.45×10^{-6}	-92.42
HGDP00127	95	2.20×10^{-5}	8	3.84×10^{-6}	-82.56
HGDP00057	110	2.56×10^{-5}	9	4.34×10^{-6}	-83.04
HGDP00056	59	1.37×10^{-5}	11	5.30×10^{-6}	-61.33
HGDP00549	71	1.66×10^{-5}	7	3.38×10^{-6}	-79.55
HGDP01009	49	1.14×10^{-5}	5	2.41×10^{-6}	-78.82
HGDP00136	111	2.58×10^{-5}	7	3.37×10^{-6}	-86.91
HGDP01075	86	1.99×10^{-5}	14	6.77×10^{-6}	-66.00
HGDP00218	58	1.34×10^{-5}	8	3.84×10^{-6}	-71.41
HGDP01298	68	1.58×10^{-5}	12	5.78×10^{-6}	-63.35
HGDP01192	59	1.37×10^{-5}	7	3.38×10^{-6}	-75.39
HGDP01225	58	1.34×10^{-5}	10	4.81×10^{-6}	-64.22
HGDP01190	90	2.09×10^{-5}	9	4.32×10^{-6}	-79.31

Table S6: Y chromosome mutation rates estimated in previous studies

Study	Mutation rate (mutations/bp/year)	95% CI	Positions Used [Mb]
Xue *et al.* [53]	10.0×10^{-10}	3.0×10^{-10} to 2.5×10^{-9}	10.15
Poznik *et al.* [54]	8.2×10^{-10}	7.2×10^{-10} to 9.2×10^{-10}	9.99
Fu *et al.* [11]	7.6×10^{-10}	6.7×10^{-10} to 8.6×10^{-10}	1.86
Karmin *et al.* [7]	7.4×10^{-10}	6.3×10^{-10} to 9.5×10^{-10}	9.83
Helgason *et al.* [36]	8.7×10^{-10}	8.0×10^{-10} to 9.4×10^{-10}	21.3
Petr *et al.* [1]	7.3×10^{-10}	6.3×10^{-10} to 8.6×10^{-10}	6.9

Table S7: Counts of positions used for BEAST analysis shown in Figure 6.

	Variable sites	Invariable sites
A	7,682	678,097
C	8,064	404,021
G	8,121	407,713
T	7,607	679,387
Total	31,474	2,169,218

Table S8: Marginal log-likelihood for the model combinations tested using the path sampling approach as implemented in BEAST2.

Clock model	Tree model	Marginal log-likelihood
Strict	Coalescent Constant Population	-3,203,344.16
Strict	Coalescent Bayesian Skyline	-3,203,333.40
Relaxed Log Normal	Coalescent Constant Population	-3,203,346.34
Relaxed Log Normal	Coalescent Bayesian Skyline	-3,203,332.62

Table S9: ESS values obtained for BEAST analysis (for parameters for which we provided a prior) shown in Figure 6.

Parameter	ESS value
posterior	24,016
likelihood	26,407
TreeHeight	9,923
clockRate	9,755
Age_Chag2	19,309
TMRCA_Modern_Humans	9,940
TMRCA_Modern_Humans_Neandertals	9,733
TMRCA_Neandertals	14,910

Table S10: Nucleotide substitution rates estimated by the BEAST bModelTest package.

Substitution rate
$r_{AC} = 0.548$
$r_{AG} = 1.853$
$r_{AT} = 0.266$
$r_{CG} = 0.933$
$r_{CT} = 1.852$
$r_{GT} = 0.548$
